# Host specificity driving genetic structure and diversity in ectoparasite populations: Coevolutionary patterns in *Apodemus* mice and their lice

**DOI:** 10.1002/ece3.4424

**Published:** 2018-10-03

**Authors:** Jana Martinů, Václav Hypša, Jan Štefka

**Affiliations:** ^1^ Biology Centre CAS Institute of Parasitology České Budějovice Czech Republic; ^2^ Faculty of Science University of South Bohemia České Budějovice Czech Republic

**Keywords:** *Apodemus*, coevolution, dispersal, genetic diversity, host specificity, *Polyplax*

## Abstract

A degree of host specificity, manifested by the processes of host–parasite cospeciations and host switches, is assumed to be a major determinant of parasites’ evolution. To understand these patterns and formulate appropriate ecological hypotheses, we need better insight into the coevolutionary processes at the intraspecific level, including the maintenance of genetic diversity and population structure of parasites and their hosts. Here, we address these questions by analyzing large‐scale molecular data on the louse *Polyplax serrata* and its hosts, mice of the genus *Apodemus*, across a broad range of European localities. Using mitochondrial DNA sequences and microsatellite data, we demonstrate the general genetic correspondence of the *Apodemus/Polyplax* system to the scenario of the postglacial recolonization of Europe, but we also show several striking discrepancies. Among the most interesting are the evolution of different degrees of host specificity in closely related louse lineages in sympatry, or decoupled population structures of the host and parasites in central Europe. We also find strong support for the prediction that parasites with narrower host specificity possess a lower level of genetic diversity and a deeper pattern of interpopulation structure as a result of limited dispersal and smaller effective population size.

## 1 INTRODUCTION

The formation and maintenance of genetic structure within populations are contingent upon an interplay of various factors, such as environment, geographic distribution, life strategy, population history. In parasites, particularly in those with life cycles closely bound to their hosts (e.g., parasitic lice), the host represents the parasite‐only environment. In such cases, parasites typically develop a strong narrow host specificity, and their population structure, diversity, and speciation processes are assumed to be strongly determined by their host.

At an interspecific level, this results in a parallel evolution, which may lead to an almost perfect fit between the host's and the parasite's phylogenies (Hughes, Kennedy, Johnson, Palma, & Page, 2007; Light & Hafner, 2008). In most cases, however, host switches blur the cophylogenetic signal, even in highly host‐specific parasites (Banks, Palma, & Paterson, 2006; Ricklefs, Fallon, & Bermingham, 2004; du Toit, Van Vuuren, Matthee, & Matthee, 2013).

Possible processes causing such incongruences have often been discussed in the parasitological literature, and a complex conceptual background has been developed (Clayton, Bush, & Johnson, 2004; Lion & Gandon, 2015; Page, 2003). For example, it has been suggested that the biogeography, social behavior, and vagility of the hosts affect the level of congruence in host–parasite equally or even to a greater extent than the physiology and life history traits of the parasite. However, estimating the degree of intimacy for a particular host–parasite association is not a simple task. It may even be counterintuitive, if previously unforeseen factors are involved in the interaction (e.g., the host abundance determining the parasite's dispersal ability; Engelbrecht, Matthee, du Toit, & Matthee, 2016). The key to understanding a coevolutionary pattern is the investigation of the parasites’ population genetics and dynamics and their main determinants. At this intraspecific level, current research has shown that parasite diversity and population structure are affected by several factors, mainly shared demographic history (Nieberding, Morand, Libois, & Michaux, 2004; Štefka, Hoeck, Keller, & Smith, 2011), host dispersal capabilities affecting parasite gene flow (McCoy, Boulinier, Tirard, & Michalakis, 2003; Štefka, Hypša, & Scholz, 2009; van Schaik, Kerth, Bruyndonckx, & Christe, 2014), and the spectrum of parasitized hosts (Archie & Ezenwa, 2011; Barrett, Thrall, Burdon, & Linde, 2009). Nadler (1995) stressed the role of host specificity, predicting that multihost parasites display a shallower population structure due to having a better chance to disperse.

Several studies on the natural populations of parasite species sharing sympatric hosts have addressed these issues, for example the coevolutionary reconstruction of feather lice species with extremely different host specificities (Johnson, Williams, Drown, Adams, & Clayton, 2002) or the investigation of two generalist pinworms from Caribbean reptiles (Falk & Perkins, 2013) or the analysis of population sizes and selection in the bacterium *Anaplasma* (Aardema & von Loewenich, 2015). These works often support Nadler's hypothesis by showing that parasites with a stronger host specificity possessed a more pronounced genetic structure. Research on a related topic using generalist flea parasites (van der Mescht, Matthee, & Matthee, 2015) suggested that the tightness of the association between a host and its parasite represents an important factor. However, while in free‐living organisms the effect of the ecological parameters and their shifts on population genetics are well explored (Lemoine et al., 2016), the extent to which even moderate changes in host specificity shape the structure and genetic diversity of parasites remains largely unknown.

In this study, we address the impact of host specificity on the genetics of parasite populations using the sucking louse *Polyplax serrata* and its hosts, mice of the genus *Apodemus*. The *Apodemus* model possesses representatives with a different geographic and ecological structure. The two most widespread species, *Apodemus flavicollis* and *A. sylvaticus,* co‐occur throughout the majority of their European distribution in sympatry or even syntopy (Darvish, Mohammadi, Ghorbani, Mahmoudi, & Dubey, 2015; Demanche et al., 2015; Michaux, Libois, & Filippucci, 2005). They separated more than 4 million years ago (mya) (Michaux & Pasquier, 1974) and responded differently to the Quaternary climatic oscillations (Michaux et al., 2005). The nonuniform evolutionary history of the two species also had an impact on the genealogies of their parasites, including endoparasitic helminths (Nieberding, Libois, Douady, Morand, & Michaux, 2005; Nieberding et al., 2004), and ectoparasites such as the sucking lice of the genus *Polyplax* (Štefka & Hypša, 2008).

The basic genetic structure of the *Polyplax*/*Apodemus* system (Štefka & Hypša, 2008) shows this system to be a useful model for studying coevolution through the analysis of population‐level codivergence and raises several interesting questions/hypotheses. At the general level, Štefka and Hypša (2008) showed that the genealogy and current geographic distribution of the lice were clearly coupled with the evolutionary history and distribution of *Apodemus* hosts. However, host specificity and phylogeographic patterns varied across three main mtDNA‐based lineages of the parasite (designated as A, B, and C in Štefka and Hypša (2008)). Two lineages, A and B, were more ubiquitous in their distribution and occurred in sympatry, but differed in their degree of host specificities. Both clades shared *A. flavicollis* as a common host and mostly occupied sympatric localities in central Europe. However, Lineage A also parasitized another species, *A. sylvaticus*, and was also found in western Europe (France and United Kingdom). Due to the differences in host specificity, in this study we refer to the two lineages as *N* (nonspecific, Lineage A) and *S* (specific, Lineage B). The lice of Lineage C inhabited mainly *A. agrarius* and *A. uralensis* occurring in the central and eastern regions of Europe, and here, we refer to it as Lineage *Aa*. Štefka and Hypša (2008) also uncovered a lineage from *A. peninsulae* from central Asia (Baikal Lake locality), hereafter referred to as the *Ape* lineage. Here, using mtDNA and multilocus data we analyze the phylogeographic and population genetic structures of an extensive sample from multiple European countries to answer the following questions: (a) Do the mtDNA *Polyplax* lineages (Štefka & Hypša, 2008) retain their integrity and host specificity if analyzed with multilocus data from considerably extended geographic sampling? (b) Do *Polyplax* parasites possess a stronger pattern of population structure compared to their hosts as a result of increased mutation rates and small effective population sizes (*N*_e_)? (c) Is host dispersal the determining factor of the parasite gene flow? That is, do the parasitic lineages *N* and *S*, with different levels of host specificity, follow Nadler's hypothesis (Nadler, 1995) in the sense of (a) deeper population structure in the more host‐specific lineage caused by lower dispersal opportunities, and (b) significant differences in genetic diversity between sympatric *N* and *S* populations?

## 2 MATERIALS AND METHODS

### 2.1 Host sampling and DNA isolation

**Figure 1:**
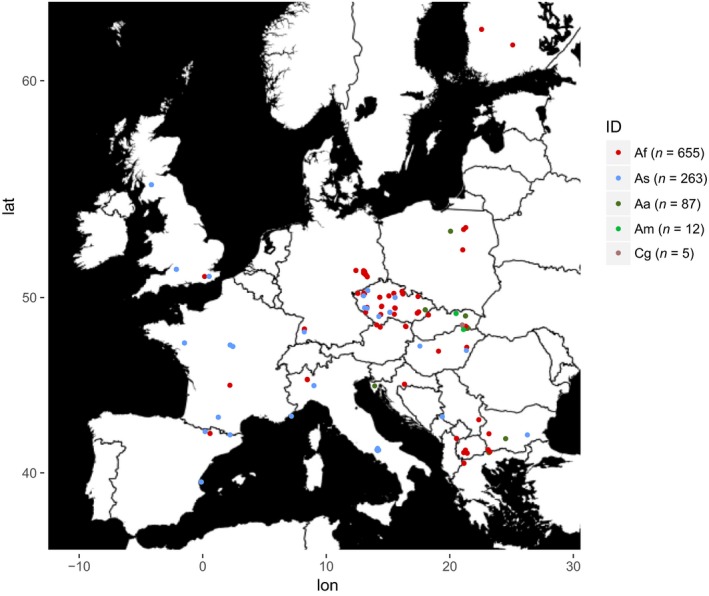
Map of sampling localities. Abbreviations: Af*—Apodemus flavicollis*, As*—A. sylvaticus*, Aa*—A. agrarius*, Am*—A. microps*, Cg*—Clethrionomys glareolus*, lat—latitude, lon—longitude

Mice were captured in wooden snap traps. *Apodemus* tissue samples (ear or fingertips) were preserved in ethanol, and the mice were examined for lice by visual checking and combing. Lice were stored in 100% ethanol in the freezer. Field studies were carried out with permits listed in the Supporting information Document S1. A total of 2,352 specimens of *Apodemus* hosts were collected across 14 European countries during the years 2005–2015. A total of 216 mice were infected with *P. serrata* resulting in a 9.18% prevalence. Host and parasite samples of infected mice and a subset of noninfected hosts covering a large part of the European continent (Figure 1, Table 1 and Supporting information Table S1) were analyzed genetically. DNA extractions were performed with a QIAamp DNA Micro Kit (Qiagen) into 30 μl of AE buffer. Louse skeletons were preserved in 70% ethanol as vouchers. Host DNA was isolated from the host tissue with a DNeasy Blood & Tissue Kit (Qiagen).

**Table 1:** List of sampling localities providing numbers of samples analyzed for each organism and marker

Country	Abbreviation	Polyplax lineage	No. of individuals analyzed per gene			Host species	No. of host individuals analyzed per gene	
			COI	Micro	Concat		D‐loop	Micro
Bulgaria	BG	Aa	3	6		*Aa*		
		N	1			*Af*	3	
Croatia	HR	Aa	4		2	*Aa*		
		S	4	4	1	*Af*	2	2
Czech Republic	CZ	Aa	18	5		*Af*	78	83
		N	44	36	1	*As*	18	15
		S	164	106	4			
Finland	FIN	–				*Af*	1	4
France	F	N	22	27	3	*Af*	7	7
		S	7	8	1	*As*	22	22
						
Germany	D	N	26	11		*Af*	55	50
		S	70	41	2	*As*	5	5
Hungary	H	–				*Af*	2	
						*As*		2
Italy	I	N	10	5	2	*Af*	7	8
		S	18	14	1	*As*	8	5
Macedonia	MK	S	51	44	2	*Af*	35	25
Poland	PL	Aa	3		1	*Af*	5	2
		N	4		2			
Russia	Ru	Ape		5	1			
Slovakia	SK	Aa	38	31		*Af*	23	5
		N	7	4		*Aa*		
		S	27	11		*Au*,* Cg*		
Serbia	Srb	N	1			*Af*		3
		S	9	4		*As*	1	2
Spain	SP	–				*As*	26	17
United Kingdom	GB	N	22	18	2	*Af*	1	
		S	3			*Af*	5	6
						*As*	17	9

Notes

Abbreviations for genetic markers: Concat: concatenated dataset (COI+ three nuclear loci); Micro: microsatellites; N: nonspecific lineage; S: specific lineage; Aa: lineage with affinity to *Apodemus agrarius*;* Af*:* Apodemus flavicollis*;* As*:* Apodemus sylvaticus*;* Aa*:* Apodemus agrarius*;* Au*:* Apodemus uralensis*;* Ape*:* Apodemus peninsulae*;* Cg*:* Clethrionomys glareolus*.

### 2.2 DNA sequencing and population analysis

A fragment of the mitochondrial cytochrome oxidase subunit I gene (COI, 379 bp) was amplified for 430 specimens of *Polyplax serrata* lice from 216 *Apodemus* hosts using primers L6625 and H7005 (Hafner et al., 1994). These primers, reliably amplifying louse DNA samples, were selected to provide a gross picture of population structure across the whole sample set. For a better understanding of the relationships among the main mtDNA lineages of lice, a longer fragment of COI (1,027 bp), together with three nuclear genes VATP21 (304 bp), hyp (380 bp), and TMEDE6 (215 bp), was obtained for selected specimens of *Polyplax* (*n* = 25), using COI primers LCO1490 and H7005 (Folmer, Black, Hoeh, Lutz, & Vrijenhoek, 1994) and nuclear primers published by Sweet, Allen, and Johnson (2014). A description of the PCR reactions, thermal cycling conditions, and sequencing is provided in Supporting information Document S1. A mitochondrial D‐loop region with the entire tRNA^Thr^, tRNA^Pro^, and the beginning of the 12S tRNA region (1,002 bp) was gained for 229 individuals of *A. flavicollis* and 92 specimens of *A. sylvaticus* with primers 1, 2bis, 3, and 4 (Bellinvia, 2004) using the PCR conditions described in Supporting information Document S1.

Obtained sequences were assembled in GENEIOUS 8.0.2 (Biomatters, Ltd), collapsed into haplotypes using ALTER (Glez‐Peña, Gómez‐Blanco, Reboiro‐Jato, Fdez‐Riverola, & Posada, 2010) and submitted to GenBank under accession numbers MH723758‐MH724187. Phylogenies were reconstructed by maximum likelihood (ML) and Bayesian inference (BI). For all analyses, the best‐fit models (listed in Supporting information Document S1) were selected according to a corrected Akaike information criterion using jModelTest2 (Darriba, Taboada, Doallo, & Posada, 2012). For the lice, *Polyplax spinulosa* was used as outgroup. For the hosts, *Apodemus sylvaticus* and *A. flavicollis* phylogenies were rooted with three individuals of the other species (three of *A. sylvaticus* with *A. flavicollis* and vice versa). Bayesian (BI) analyses conducted in MrBayes 3.2.4 (Ronquist et al., 2012) consisted of two parallel Markov chain Monte Carlo simulations with four chains run for 10 million generations with sampling frequency of 1,000 generations. The convergence of parameter estimates and their ESS values was checked in software TRACER 1.6 (Rambaut, Drummond, Xie, Baele, & Suchard, 2018). Two and a half million generations (25%) were discarded as burn‐in. Maximum likelihood analyses were computed using PhyML 3.0 (Guindon et al., 2010) with 1000 bootstrap replicates to obtain nodal support.

To explore population genetic patterns and compare them with phylogeny derived results, we reconstructed haplotype networks, calculated standard diversity measures, and performed hierarchical AMOVA as detailed in Supporting information Document S1.

### 2.3 Microsatellite genotyping and population structure

To analyze population structure and level of diversity in individual populations of the parasite and two of its hosts, microsatellite loci were incorporated into the study. For 380 individuals of *Polyplax serrata* included into the mtDNA analysis, sixteen microsatellite loci were amplified in four multiplex PCR assays developed by Martinů et al. (2015). All microsatellite loci were tested for departure from the Hardy–Weinberg equilibrium (HWE) and linkage disequilibrium (LD) between loci pairs for all populations (with *n* ≥ 5 individuals) in GenAlEx 6.5 (Peakall & Smouse, 2012). Micro‐checker 2.2.3 was used to evaluate whether the observed heterozygote deficiencies could be explained by the occurrence of null alleles (Van Oosterhout, Hutchinson, Wills, & Shipley, 2004). For *Apodemus flavicollis* and *A. sylvaticus,* seven microsatellite loci were amplified in two multiplex assays, following Harr, Musolf, and Gerlach (2000) and Aurelle et al. (2010).The additional five loci exclusively specific to *A. flavicollis*, using multiplexes according to Aurelle et al. (2010), and 10 loci exclusively specific to *A. sylvaticus* (Harr et al., 2000) were amplified to complement datasets of each species. Altogether, 229 individuals of *A. flavicollis* and 92 individuals of *A. sylvaticus* were genotyped and all sampled specimens were also included in the mtDNA phylogenies. All loci were tested for departure from HWE and for LD between pairs of loci in GenAlEx 6.5 (Peakall & Smouse, 2012).

To determine whether populations of the parasite belonging to the *S*,* N*,* Aa*, and *Ape* mtDNA lineages form matching clusters in their nuclear data, or whether they admix, the multivariate technique of principal coordinate analysis (PCoA) was computed from the genetic distance matrix calculated across multiple loci for each pair of individuals. The same analysis was performed also on the population level. PCoA together with an assignment test of *S* and *N* lineages was performed in GenAlEx 6.5 (Peakall & Smouse, 2012). The PCoA as described above for *Polyplax* was performed also for both *Apodemus* species to reconstruct their population structure and to reveal the level of integrity/mixing of individual mtDNA lineages within and between populations. PCoA‐based picture of population structure was checked using other distance‐based methods and Bayesian clustering methods described in Supporting information Document S1 in detail.

### 2.4 Distribution of genetic diversity in *Polyplax* and *Apodemus*

To assess the influence of geographic distance on genetic relatedness, Mantel tests (Mantel, 1967) were used to test for isolation by distance (IBD) using microsatellite estimates of genetic differentiation (*F*_ST_, *G*_ST_, and *D*_JOST_) and geographic distances separately for both *Polyplax* lineages and both *Apodemus* species in the R package adegenet (Jombart, 2008). Statistical significance was computed by 10,000 random permutations. Because the effect of IBD may play different roles at different geographic scales, we analyzed the spatial autocorrelation coefficient (*r*) for *Polyplax S* and *N* lineages and both *Apodemus* hosts. The analyses were performed in GenAlEx 6.5 (Peakall & Smouse, 2012), where *r* was calculated for increasing distance classes with a 95% confidence interval obtained by 1,000 bootstrap replicates and 10 000 permuted *r* values (Peakall, Ruibal, & Lindenmayer, 2003; Smouse & Peakall, 1999).

The impact of host genealogy on the genetic structure of the parasite was evaluated by correlating the *F*_ST_ (and *G*_ST_) matrixes of each of the *Polyplax* lineages and its host species using Mantel tests in R package adegenet and GenAlEx 6.5 (Jombart, 2008; Peakall & Smouse, 2012).

To determine the possible impact of host width (specificity) on population diversity of the parasites, we analyzed differences in the level of genetic diversity between *S* and *N* lineages of *Polyplax* using microsatellite data. *F*_ST_ and gene diversity (*H*) indices were calculated for pairs for *S* and *N* populations that were collected at identical sites (sympatric populations) or at closely placed sites (within 30 km from each other). Seven population pairs from five European countries matched these criteria and contained a sufficient number of genotyped individuals (*n* > 3). *F*_ST_ calculations were performed in FSTAT 2.9.3.2 (Goudet 2002) with *p*‐values determined by 10,000 permutations. *H* estimates were obtained in GenAlEx 6.5 (Peakall & Smouse, 2012).

## 3 RESULTS

### 3.1 Phylogeny of *Polyplax serrata* and the *Apodemus* species

**Figure 2:**
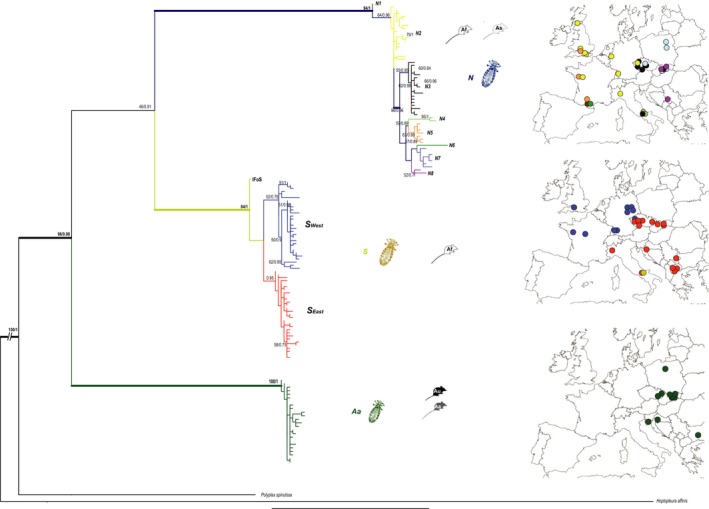
Mitochondrial DNA phylogeny for 556 specimens of *Polyplax serrata*. Maximum likelihood phylogeny was obtained with PHYML, statistical support (ML bootstrap/Bayesian posterior probability) is provided above nodes, supported clades (ML bootstrap higher than 80%/Bayesian posterior probability above 0.95) in bold. Geographic distribution of Subclades *N* and *S* is provided using matching colors. Abbreviations of clades and host species: *N*—nonspecific clade; *S*—specific clade; *S*_West—_western lineage of specific clade; *S*_East_—eastern lineage of specific clade; *Aa*—*Apodemus agrarius* and *uralensis* clade; Aa—*A. agrarius*; Af—*A. flavicollis*; As—*A. sylvaticus*; Au—*A. uralensis*

Partial COI genes were sequenced for 430 louse specimens and aligned with 126 sequences obtained by Štefka and Hypša (2008). Final mitochondrial dataset contained sequences of 556 *Polyplax* specimens (Table 1 and Supporting information Table S1). Phylogenetic analyses of the short matrix (379 bp, 138 haplotypes) clustered the lice into three well‐supported lineages (Figure 2) described previously by Štefka and Hypša (2008).

The *S* and *N* lineages were found in sympatry or at adjacent localities across a large geographic area (Figure 2). However, while the *N* lineage did not show any clear geography dependent structure, an intriguing geographic pattern was detected for the *S* lineage. This lineage split into two well‐supported subgroups with different, almost exclusive geographic distributions (except for a narrow overlap). These two subgroups are therefore designated as *Specific East* (*S*_East_) and *Specific West* (*S*_West_). The third main lineage (*Aa*) was only found in the eastern part of Europe, concurrently with its primary hosts (*A. agrarius* and *A. uralensis*).

The relationships between the *N*,* S,* and *Aa* lineages were not well supported in the analysis of short COI sequences, but could be reliably established by analyzing 25 representative samples for which longer COI sequences (1,027 bp) were concatenated with three nuclear genes. This analysis clustered the *S* and *N* lineages as sister groups (Supporting information Figure S1).

**Figure 3:**
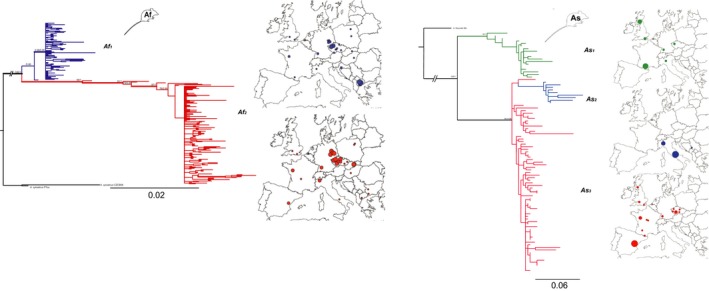
Mitochondrial DNA phylogeny for 229 specimens of *Apodemus flavicollis* and 92 specimens of *Apodemus sylvaticus*. Maximum likelihood phylogeny was obtained with PHYML, statistical support (ML bootstrap higher than 50% Bayesian posterior probability above 0.6) is provided above nodes, supported clades (ML bootstrap higher than 80%/Bayesian posterior probability above 0.95) in bold. Geographic distribution of subclades *Af*_1_ *, Af*_2_ *, As*_1_ *, As*_2_, and *As*_3_ is provided using matching colors

For the host, we obtained D‐loop sequences from 229 *A*. *flavicollis* and 92 *A. sylvaticus* samples. *A. flavicollis* phylogeny revealed two phylogenetically distinct clusters (*Af*_1_ and *Af*_2_) largely overlapping in their geographic distribution (Figure 3) but differing in their abundance. For *A. sylvaticus*, phylogenetic tree contained three clusters (Figure 3). Two of them, *As*_1_ and *As*_3_, overlapped in their distributions across western Europe; however, *As*_3_ was found more frequently across the whole area and extended also to central Europe and the Iberian Peninsula. *As*_3_ was paraphyletic with respect to the third lineage, the Italian‐Balkan clade *As*_2_.

Genetic differentiation between the western and southeastern samples of the lice demonstrated by the phylogenetic trees (Figure 2 and Supporting information Figure S1) and haplotype networks (Supporting information Figure S2) is in accord with the nucleotide diversity statistics (Supporting information Tables S2 and S3), suggesting a recent spread of *Polyplax* populations from glacial refugia, accompanied by population decline and subsequent expansion in several lineages. The demographic pattern in the hosts was less pronounced. Haplotypes belonging to major clades within *A. flavicollis* and *A. sylvaticus* were geographically admixed, high levels of haplotype diversities were obtained for lineages within both species, and fewer cases of past demographic fluctuations were revealed (Supporting information Figures S5 and S6, and Supporting information Tables S2 and S3).

### 3.2 Microsatellite diversity and structure in the *Polyplax‐Apodemus* system

The overall microsatellite diversity obtained for parasite and host samples are summarized in Table 2; Supporting information Table S5 and S8. For the lice, each of the microsatellite loci was polymorphic in at least 15 of the 32 populations, with up to 11 alleles per locus and population (Supporting information Table S5). Correspondingly to the low average heterozygosity (*H*_e_, Table 2), all louse populations showed significant deviations from the Hardy–Weinberg equilibrium due to heterozygote deficiencies in at least one locus, but none of the loci was out of HWE across all populations (Supporting information Table S6). The deviations were more frequent in the *S* lineage than in the *N* lineage. Micro‐checker analysis indicated possible occurrence of null alleles in several cases; however, adjusted estimates of gene diversity of few populations differed only marginally (Supporting information Table S7), and we thus decided to keep all data for the subsequent analyses. Pairwise *F*_ST_ values indicated considerable degree of genetic differentiation between populations (with *n* ≥ 5), ranging from 0.04 to 0.65 in the *S* lineage and 0.10 to 0.39 in the *N* lineage (Supporting information Table S8).

**Table 2:** Observed and expected heterozygosities for populations of *Polyplax serrata S*,* N* lineages, *Apodemus flavicollis*, and *A. sylvaticus*

Pop	*PS S* lineage		*PS N* lineage		*A. flavicollis*		*A. sylvaticus*	
	*H*_o_	*H*_e_	*H*_o_	*H*_e_	*H*_o_	*H*_e_	*H*_o_	*H*_e_
CZBen	0.131	0.417			0.667	0.552		
CZCB			0.484	0.495				
CZCM1	0.072	0.162	0.435	0.569	0.563	0.622		
CZDou	0.219	0.285			0.583	0.709		
CZJach	0.200	0.256						
CZLi05	0.229	0.481	0.348	0.383	0.638	0.818		
CZPl			0.323	0.508			0.571	0.679
CZStr	0.199	0.299	0.354	0.465	0.670	0.763		
CZVyk	0.202	0.335						
DBa	0.353	0.420	0.335	0.554	0.600	0.738	0.718	0.729
DKot	0.050	0.073			0.625	0.630		
DKrei					0.741	0.741		
DLau	0.088	0.181			0.660	0.752		
DPin					0.604	0.641		
DSol	0.161	0.274			0.735	0.722		
DTor	0.218	0.269			0.740	0.734		
EBa							0.687	0.848
FGu	0.110	0.348	0.472	0.608	0.639	0.767	0.574	0.631
FTou			0.451	0.545			0.638	0.793
Fin					0.542	0.503		
GBAs			0.343	0.459			0.750	0.664
GBSc			0.250	0.297				
GBSt			0.539	0.625			0.594	0.663
HRVS	0.328	0.363						
IBri	0.174	0.403			0.668	0.748		
IBu			0.396	0.405				
ICiS			0.500	0.477				
MK8	0.425	0.602						
MK9	0.436	0.672			0.732	0.809		
MK10	0.469	0.636			0.764	0.799		
MK12	0.000	0.455						
PLPu			0.141	0.373				
SKPo	0.136	0.174						
SKRuz			0.422	0.547				
SrbSP	0.141	0.324						
Average	0.207	0.354	0.402	0.492	0.657	0.709	0.647	0.715

*Note*

Population abbreviations as in Supporting information Table S1.

In the hosts, *A. flavicollis* and *A. sylvaticus,* the number of alleles per locus varied from one to 15 alleles with an average of four alleles per locus and population (Supporting information Table S9). In *A. flavicollis*, for which 12 loci were analyzed, two populations were in HWE, the rest showed deviations from HWE in one to four loci, and the German population DLau had six loci of HWE (Supporting information Table S10). In *A. sylvaticus*, with 17 loci analyzed, the British population GBA showed no deviations from HWE, the majority of other populations had one to four loci of HWE, the French population FTou had five loci, and the Spanish population EBa had 11 loci of HWE. Pairwise *F*_ST_ values showed considerable genetic structure, ranging from 0.03 to 0.47 in *A. flavicollis* and 0.04 to 0.59 in *A. sylvaticus* (Supporting information Table S8).

**Figure 4:**
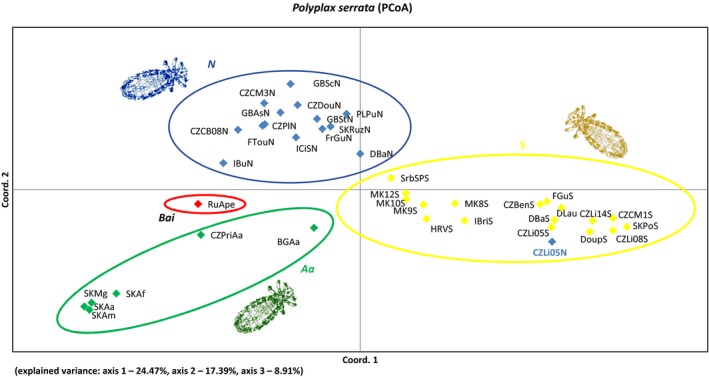
Principal coordinate analysis (PCoA) of *Polyplax serrata* populations using microsatellite data. Colors match major lineages used in Figure 2. Population sample containing mtDNA introgressed from the N lineage (CZLi05N) is highlighted in blue. Population abbreviations as in Supporting information Table S1

PCoA of the microsatellite datasets revealed deep genetic structure in the parasite and, on the contrary, a relatively shallow divergence in the hosts. In *Polyplax,* the analysis divided the populations into clusters corresponding to the main mtDNA lineages (Figure 4). The only discrepancy was found for the Czech population Litvínov (CZLi05N; blue in Figure 4), which belongs to the *N* lineage according to the mtDNA data, but clusters together with *S* populations in the microsatellite analysis. Genetic differentiation between the *S* and *N* lineages was also obvious from the assignment test performed in GenAlEx (results not shown) and from the Bayesian and distance‐based clustering (Supporting information Figures S8 and S9). On the intralineage level, PCoA of individuals from *S* and *N* lineages showed in most cases that lice sampled from the same locality formed compact structures, and geographically close populations often showed genetic proximity (Supporting information Figure S10a, b). This trend was more pronounced in the *S* lineage compared to the *N*. PCoA based on data for the whole populations revealed further differences between the *S* and *N* lineages (Supporting information Figure S10c, d). While within *S* lineage the populations clearly clustered according to their geographic origin, a fractional geographic clustering was also discernible in the *N* lineage, but it did not create such explicit clusters as in the *S* lineage.

For the hosts, analyses performed on a set of seven microsatellite loci shared by both host species (PCoA, Bayesian and distance‐based clustering—Document S1) agreed with the mtDNA pattern confirming that *A. flavicollis* and *A. sylvaticus* represent two separated species. On the intraspecific level, despite analyzing more loci, the PCoA results demonstrated in both species that host individuals from different mtDNA subclades did not form separated clusters when retrieved from sympatric localities (Supporting information Figure S11a, b). Geographically delimited populations (localities) were more admixed than in the parasites and did not cluster together. On the population level, PCoA (Supporting information Figure S11c, d) showed formation of several genetic lineages, which, however, did not correspond to the mtDNA genealogy and showed only a limited correspondence to geography (e.g., GB and FR populations in *A. sylvaticus,* Supporting information Figure S11d). Similar results were obtained also from the Bayesian and distance‐based clustering analyses (Document S1, Supporting information Figures S9 and S12).

### 3.3 Spatial structure of the parasites and hosts

**Figure 5:**
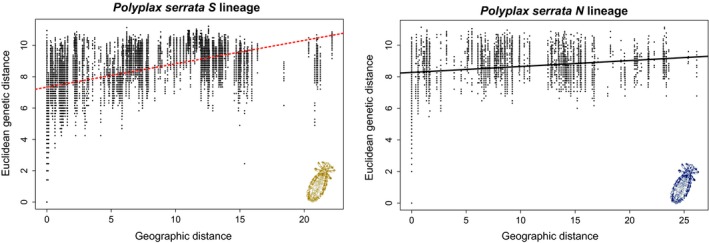
Correlation between Euclidean genetic distances and geographic distances for pairs of *Polyplax serrata* individuals. Plots were generated separately for S and N lineages in adegenet. Correlation was significant (red dashed line) for the S lineage and nonsignificant (black line) for the N lineage (10,000 permutations)

Correlations between genetic pairwise matrices and geographic distances, as analyzed by Mantel tests, varied in dependence on both the species/lineage of the host/parasite and the exact statistics used. *F*_ST_ tests found significant IBD only within *A. sylvaticus* (Supporting information Figure S13). *G*_ST_ tests were statistically significant for *Polyplax S* lineage (Supporting information Figure S14) and for *A. sylvaticus* (Supporting information Figure S13), whereas *D*_JOST_ test was significant only for the *Polyplax S* lineage (Supporting information Figure S14). When assessed as the correlation between Euclidean distances (performed on the level of individuals) and geographic distances, the IBD was only significant for the *S* lineage, with a markedly larger correlation than for the *N* lineage (Figure 5).

The autocorrelation coefficient (*r*), used to evaluate the effect of IBD on different geographic scales, revealed in all evaluated organisms (*Polyplax* lineages *S* and *N*,* A. sylvaticus,* and *A. flavicollis*; Supporting information Figure S15) a positive significant autocorrelation, which was declining with increase in the distance between populations. This pattern indicates that IBD is strongest between the neighboring populations in both hosts and parasites. However, the spatial extent and the strength of the autocorrelation differed between organisms, showing stronger signal at short distances for the parasite compared to the hosts. The highest values of autocorrelation coefficient (*r)* in *Polyplax* lineages were two times greater than those of the hosts. In the hosts, the *r* value was 10 times lower at the shortest distance range in *A. flavicollis* than in *A. sylvaticus*, which corresponded with the nonsignificant results of Mantel tests in *A. flavicollis*.

### 3.4 Differences in population diversities between *S* and *N* lineages of Polyplax

**Figure 6:**
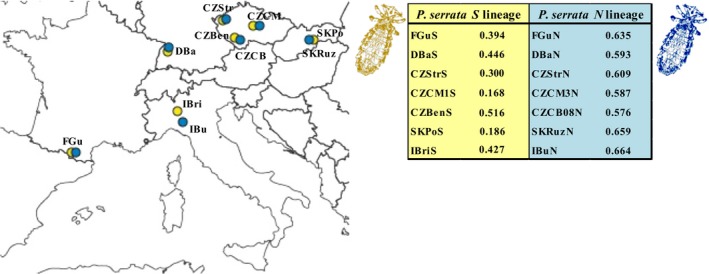
Gene diversity (*H*) and geographic distribution for seven pairs of sympatric *S* and *N* lineage populations of *Polyplax serrata*. Color codes as in Figure 2. Population abbreviations as in Supporting information Table S1

Microsatellite data were used to verify Nadler's hypothesis using populations of the *S* and *N* lineages as representatives of the specialist and generalist parasitic strategies. According to the prediction, *F*_ST_ and *H* indices calculated for each of the two lineages revealed a lower genetic diversity and a stronger population structure for the *S* lineage. The *F*_ST_ index was statistically lower for the *N* lineage (0.241) than for the *S *lineage (0.460) (15 000 permutations). On the contrary, the *H* index was markedly higher for populations of the *N* lineage (0.587) than for the *S* populations (0.389) (15,000 permutations). A more detailed study of both lineages performed on seven pairs of sympatric (or closely located populations) showed, in all pairwise comparisons, higher values of *H* for *N* populations than for *S* (Figure 6).

## 4 DISCUSSION

Using the *Apodemus*/*Polyplax* model, we demonstrate that coevolutionary processes, when viewed from a broad‐scale population perspective, may produce surprisingly complex and intriguing patterns (Figures 2 and 3). At the most general level, the obtained patterns conform to the traditionally held views that parasites phylogenies and genealogies are strongly determined by their hosts and that populations of parasites have a lower genetic connectivity and are more structured than those of the hosts (Engelbrecht et al., 2016; Koop, DeMatteo, Parker, & Whiteman, 2014; Nieberding & Olivieri, 2007). However, at a more subtle level, the structure, genetic diversity, and host specificity of the parasite populations differ even between closely related sister clades. For example, although the two main sister lineages of the parasite (*S* and *N*) are widely distributed and share an identical host, *A. flavicollis*, only the *S* lineage is strictly specific, while lice of the *N* lineage can also be found on the other host species, *A. sylvaticus*. As the specific and nonspecific samples were collected in sympatry, sometimes even from identical host individuals, we suppose that the absence of the *S* lineage on *A. sylvaticus* is due to adaptive constraints rather than lack of opportunity to switch hosts. However, the most striking instance of the observed irregularities is probably provided by the sharp difference seen in the postglacial colonization process between *A. flavicollis* and its specific parasite, the *S* lineage of *Polyplax*. In this host/parasite association, the encounter of populations from different refugia resulted in a largely admixed European population of the host, while the louse populations remained genetically separated, with only a narrow contact zone (discussed below). This remarkable complexity of the whole system is further increased by various unique genetic events, such as a mitochondrial introgression of the *N* louse clade into a single population of the other clade (e.g., Figure 4). At last, we demonstrated that the effect of the level of host specificity on population structure and diversity of ectoparasite populations follows Nadler's predictions. We document this by a comparison between the specific lineage *S*, with low genetic diversity and a higher level of isolation by distance between its populations, and the more generalist *N* lineage found on two host species (Figure 5 and Supporting information Figure S14).

### 4.1 Decoupled process of postglacial recolonization in host and parasite populations

The observed distribution of the clades and haplotypes within the *Apodemus/Polyplax* system corresponds in general to the presumed (re)colonization processes of Europe, determined by the biogeographic and climatic changes of the Quaternary glaciation. The host species likely recolonized Europe from several refugia (Russian Ukrainian and Balkan for *A. flavicollis,* Iberian peninsula/southern France for *A. sylvaticus*) and formed panmictic populations covering most of the territory of European (Figure 3; Supporting information Figures S5 and S6). It is interesting that while the lice accompanied the two host species during their retreat to refugia and subsequent expansion, they have not mirrored straightforwardly their recolonization process. A striking discrepancy was detected between the distribution of the *A. flavicollis* mtDNA lineages (Supporting information Figure S5) and the *A. flavicollis* specific lice (*S* lineage) (Supporting information Figure S3). As shown in the Supporting information Figures S3 and S5, after their expansion from different refugia, the two mtDNA lineages of *A. flavicollis* spread across the whole sampled area and can be now be found in sympatry at identical localities. Multilocus analyses show that this secondary postglacial encounter has been followed by frequent gene flow, resulting in (re)constitution of a single highly admixed population (Supporting information Figure S12). In contrast, the two mtDNA haplotype clusters (*S*_East_ and *S*_West_) of the *P. serrata S* lineage stopped their expansion from the glacial refugia at the narrow contact zone in central Europe (Supporting information Figure S3). This incongruence is unexpected, as due to their intimate relationship, lice and their hosts are expected to share identical patterns of geographic expansion, unless the association is disrupted by a host switch. In other words, the geographic distribution of a louse species/population is believed to be entirely determined by the host(s) (Marshall, 1981). The incapability of the two louse populations to cross the contact zone thus indicates that factors other than host‐mediated distribution, or a mere within‐refugia speciation, have played a role during the recolonization process. Based on the presented data, it is difficult to hypothesize on the probable cause of this discrepancy. However, an interesting possibility is presented by the symbiotic bacteria known to inhabit the lice (Hypša & Křížek, 2007; Říhová, Nováková, Husník, & Hypša, 2017). The viability and/or reproduction of many blood feeding insects depend on various bacterial symbionts, and the intimacy of the host–symbiont association in such cases results in a metabolic cooperation between their genomes (Kirkness et al., 2010; Snyder & Rio, 2013). The long‐term isolation in refugia (potentially lasting 0.4 to 0.6 My, see Michaux, Libois, Paradis, & Filippucci, 2004) could thus lead to specific louse‐genome vs. symbiont‐genome adaptations that prevent an “incorrect” genome–genome combination.

### 4.2 Different level of resolution in mitochondrial and microsatellite data

In contrast to the mtDNA, microsatellites did not show any apparent suture between the *S*_West_ populations on the one hand and the *S*_East_ populations on the other hand. As the mtDNA‐based picture is based on extensive sampling and is well supported (Figure 2 and Supporting information Figure S3), this discrepancy may reflect the different level of historical information preserved in the microsatellite data. As shown in Supporting information Figures S9 and S10, based on the microsatellite‐derived signal, the analyses were able to recognize and cluster together geographically proximate populations, but did not provide information on the higher hierarchical structure across Europe. This picture is not entirely surprising. Due to a smaller *N*_e_ and quicker coalescence compared to nuclear loci, mtDNA is considered to be the leading indicator of speciation processes (Zink & Barrowclough, 2008). Instead, the mitonuclear discrepancy may also be the result of a selection caused by different lineages of a bacterial endosymbiont, as was shown in other insects (Kodandaramaiah, Simonsen, Bromilow, Wahlberg, & Sperling, 2013). Nevertheless, microsatellites could in future provide an appropriate tool for quantifying the volume of gene flow across the contact zone, after it is sampled more densely than in our current dataset.

### 4.3 Occasional mitochondrial introgression

Apart from this general difference, we also observed rare assignment discrepancies between the two types of data for the CZLi louse population of *A. flavicollis*. Approximately half of the specimens sampled in 2005 (CZLi05N) clustered within the *N* lineage according to mtDNA (clustered with Subclade N2 in Figure 2), whereas microsatellites placed the whole sample CZLi05 within the S lineage (Figure 4 and Supporting information Figure S10a, c). The rest of the population sample (CZLi05S) was placed within the *S* lineage by both mtDNA (cluster *S*_WEST_ in Figure 2) and microsatellites (Figure 4; Supporting information Figure S10a, c). Such discrepancies are usually explained either by the incomplete sorting of an ancestral polymorphism or by introgression after a secondary contact (Hochkirch, 2013; Toews & Brelsford, 2012). As we only found a single instance of such shared haplotypes between the two louse lineages across the whole dataset, and the repeated sampling at the locality in 2008 and 2014 did not reveal any shared haplotypes, we conclude that a recent and short‐lived mitochondrial introgression from the *N* lineage to the *S* lineage provides a more plausible explanation. Such a dynamic development, where genetic information is quickly lost (or fixed) after introgression, is in agreement with the biology of louse populations. Small, fragmented populations of lice are prone to rapid changes in their size and genetic composition. It was also demonstrated that after several generations of backcrossing, it is often difficult to trace introgression using microsatellites, and genomic tools allowing extensive screening of the genome are required (Oliveira et al., 2015).

It has recently been demonstrated in different systems that species boundaries may not be as resistant to the gene flow of either mtDNA or nuclear DNA as previously thought (Harrison & Larson, 2014). Although mitochondrial introgressions occurring together with a very low or even zero introgression of nuclear genes are rare, they were shown to occasionally happen, for example in Galapagos mockingbirds (Nietlisbach et al., 2013) and North American chipmunks (Good, Vanderpool, Keeble, & Bi, 2015). Because the *N*_e_ of mtDNA genes is four times lower than of autosomal genes, genetic drift influences mitochondrial haplotypes to a larger extent and can lead to a faster fixation of unoriginal mitochondrial haplotypes (Funk & Omland, 2003; Zink & Barrowclough, 2008). Parasites without free‐living stages and intermediate hosts generally possess a female‐biased sex ratio (Criscione, Poulin, & Blouin, 2005), which can also affect the introgression process after contact. By accident, a female‐biased sex ratio was also found in a related louse species, the *Polyplax arvicanthis* lice from the South African *Rhabdomys* (Matthee et al., 2007).

### 4.4 Host specificity governs parasite dispersal and population size: test of the Nadler's hypothesis

The dispersal capacity of parasites is to a great extent influenced by host sociality and vagility (Criscione et al., 2005; Mazé‐Guilmo, Blanchet, Mccoy, & Loot, 2016; van Schaik et al., 2014). As parasitic lice inhabit a single host during their entire life cycle, their opportunities to spread are limited to direct host contact or to shared host shelters (Marshall, 1981). Likewise, populations of host‐specific ectoparasites were recently shown to be more genetically fragmented than their hosts (Harper, Spradling, Demastes, & Calhoun, 2015; Koop et al., 2014). When comparing the dispersal activities of sucking lice and their hosts, one should expect a higher level of historical gene flow in mice and a lower level for lice because of the life history traits of the parasites, such as the lack of other vectors and occasional “missing the boat” events during the host's migration (Clayton et al., 2004; Page, 2003). In our system, we found markedly higher values of autocorrelation coefficients for both *Polyplax* lineages compared with *Apodemus* hosts, especially over shorter distances (Supporting information Figure S15), which is consistent with the expected lower level of gene flow in the parasite. Furthermore, the high rate of *H*_e_ deficiency in louse populations (Table 2) indicates that the gene flow is limited even within a single host population among the lice from different host individuals. This is in agreement with earlier reports (Harper et al., 2015; Koop et al., 2014) and supports our expectations that host dispersal is the general factor driving parasite gene flow.

In contrast to the general pattern of a more pronounced population structure in the parasite compared to its host, a lower level of differentiation in the parasites was reported by du Toit et al. (2013) in the system of *Rhabdomys* mice and *Polyplax arvicanthis* lice in South Africa. As revealed by the authors, two factors seem to have caused the discrepancy. First, the *Rhabdomys* hosts comprise four species with a parapatric distribution, forming narrow contact zones, which allow occasional host switching followed by genetic admixture of the parasites. Second, *P. arvicanthis* has approximately five times higher prevalence (60%) than *P. serrata*, and thus reaches a high *N*_e_ potentially slowing down the rate of differentiation between populations. On the contrary, despite the fact that the sympatric occurrence of *A. sylvaticus* and *A. flavicollis* should allow for a higher rate of host switching in *Apodemus* parasites than in the case of *Rhabdomys*, the evolutionarily old origin of the *S* and *N* lineages and their long‐term separation in different refugia led to an accumulation of changes that prevents successful host switching in the *S* lineage. The *N* and *S* lineages of *P. serrata* diverged ~1.5 mya (Štefka & Hypša, 2008), and their hosts were isolated in several refugia, some of them specific to only a single species, some of them shared (Michaux et al., 2004). Furthermore, the relatively low prevalence of the *P. serrata* (9%) results in small *N*_e_ that accelerates genetic drift and fragmentation of the populations.

In addition to the differences in gene flow between the hosts and the parasites, our system provided a unique opportunity to test specific predictions of Nadler's hypothesis (Nadler, 1995) by a comparison of two closely related parasites with different degrees of host specificity. According to the hypothesis, the less specialized *N* lineage should experience a higher degree of gene flow than the strictly specific *S* lineage, due to having more opportunities to find suitable hosts and hence a stronger dispersion capability. In agreement with this expectation, our IBD analysis of genetic and geographic distances among individual lice detected a steeper and statistically significant correlation in the *S* lineage in contrast to a weak and nonsignificant dependence in the *N* lineage (Figure 5).

Yet, another piece of evidence corroborating Nadler's hypothesis was provided by the comparison of genetic diversities between sympatric populations of the two louse lineages. In an overall statistical analysis, the *N* lineage populations showed a significantly lower *F*_ST_ index indicating that the *S* lineage lice (specialists) have a smaller *N*_e_ and more fragmented populations, expressed by the low frequency of heterozygotes as a result of the Wahlund effect. More important, the comparison of gene diversities between seven sympatric pairs of *N* and *S *populations (Figure 6) reached the same conclusions as the indexes calculated for the whole lineages. This multiple population comparison provides a strong body of evidence that even a moderate shift in host specificity translates into significant differences in genetic characteristics of parasite populations.

## 5 CONCLUSION

The evolutionary history of the *Apodemus–Polyplax* association across a large area of Europe is more complicated that could be expected for such a “simple” relationship between a host and its permanent ectoparasite. The traditional coevolutionary view, holding that the distribution and genetic structure of a parasite populations are determined by host phylogeography, is here reflected by the overall genetic structure of the parasite, which corresponds to the presumed (re)colonization processes of the *Apodemus* species in Europe. This, however, is not a complete picture. Some of the patterns indicate that even a strong population structure and changes in the genetic background of the parasite's populations may be driven by forces independent of the host(s). This finding warns us against simplifying tendencies when studying host–parasite coevolution and underestimation of intrinsic genetic processes in parasitic organisms. To show this, we generated and analyzed the largest and most complex body of molecular data (mitochondrial haplotypes and microsatellites) available on this host–parasite association. This also allowed us to address in detail several other issues, such as Nadler's hypothesis for parasite genetic diversity or genetic introgression in temporal parasite populations.

## CONFLICT OF INTEREST

None declared.

## AUTHOR CONTRIBUTIONS

This study forms part of the PhD research of J.M., who performed laboratory and data analyses under the supervision of J.Š., with V.H. and J.Š. conceiving the study of *Apodemus/Polyplax* coevolution. All three authors contributed toward the design of the study and drafted the manuscript.

## DATA ACCESSIBILITY

DNA sequences obtained in the frame of the study will be submitted to GenBank upon acceptance of the MS. DNA alignments and microsatellite datasets are submitted to Dryad database ( https://doi.org/10.5061/dryad.5jh39).
