# Supplementary material for: Host specificity driving genetic structure and diversity in ectoparasite populations: Coevolutionary patterns in Apodemus mice and their lice
Source: Ecol Evol. 2018 Oct 3;8(20):10008–22. doi: 10.1002/ece3.4424 (PMC6206178; doi:10.1002/ece3.4424)
Supplement: Supplementary file 1 [file ECE3-8-10008-s001.pdf]

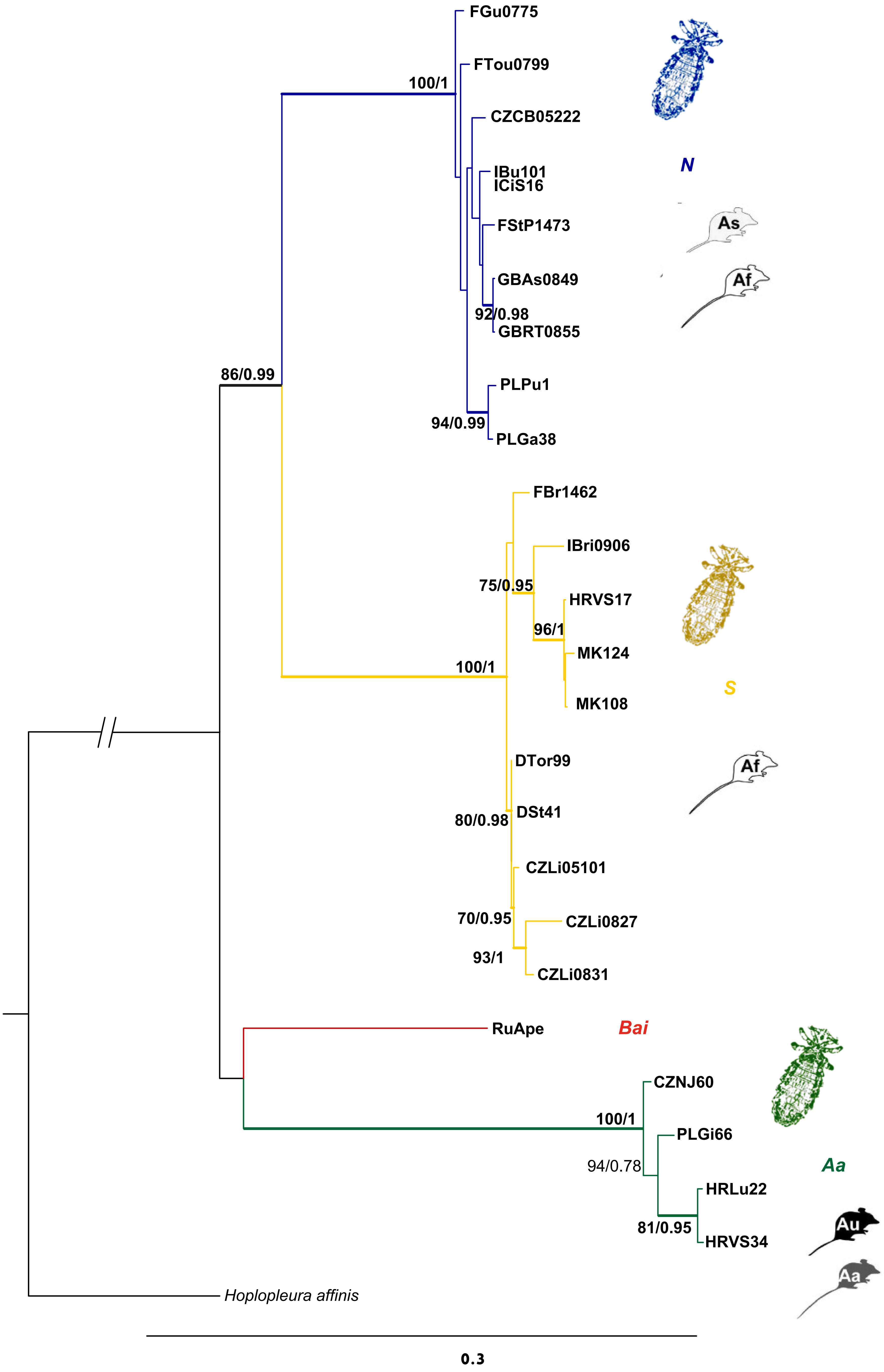

Figure S1: Molecular phylogeny of major *Polyplax serrata* clades based on a concatenated dataset of four genes (COI, VATP21, hyp and TMEDE6). Maximum Likelihood phylogeny was obtained with PHYML, statistical support (ML bootstrap higher than 50%/Bayesian posterior probability above 0.6) is provided above nodes, supported clades (ML bootstrap higher than 80%/Bayesian posterior probability above 0.95) in bold. Abbreviations as in Table S1.
