# Supplementary material for: Host specificity driving genetic structure and diversity in ectoparasite populations: Coevolutionary patterns in Apodemus mice and their lice
Source: Ecol Evol. 2018 Oct 3;8(20):10008–22. doi: 10.1002/ece3.4424 (PMC6206178; doi:10.1002/ece3.4424)
Supplement: Supplementary file 2 [file ECE3-8-10008-s002.pdf]

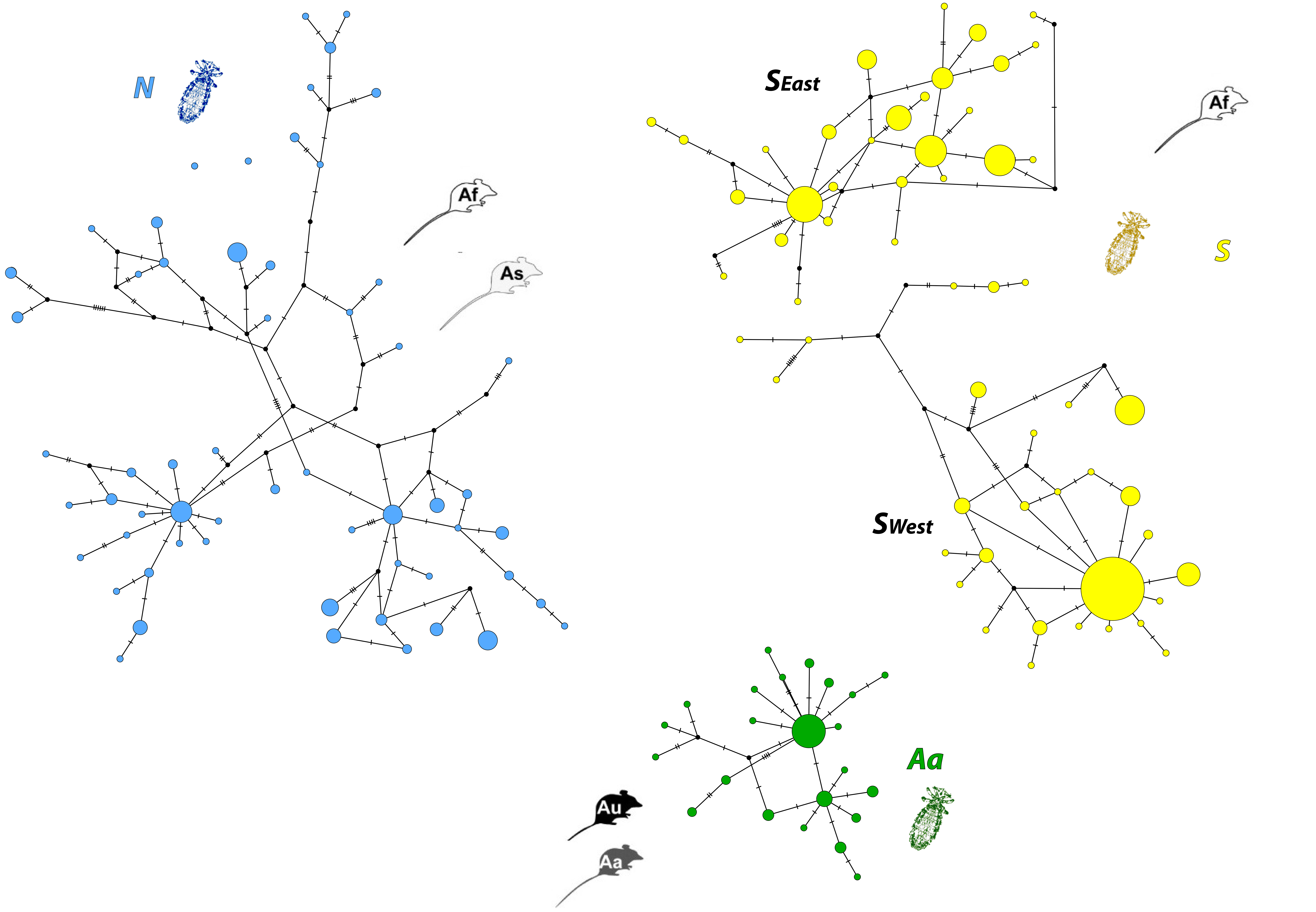

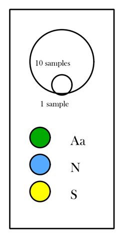

Figure S2: Haplotype networks for European lineages of *Polyplax serrata*. Networks were obtained in TCS program implemented in PopArt software using 381bp fragments of the COI gene. *N* – nonspecific lineage; *S* – specific lineage; *Aa* – lineage from *A. agrarius* and *A. uralensis*; host species abbreviations: Af – *Apodemus flavicollis*, As – *A. sylvaticus*, Aa – *A. agrarius*, Au – *Apodemus uralensis*; *SEast* and *SWest* – eastern and western clades of the *S* lineage, respectively.
