# Supplementary material for: Host specificity driving genetic structure and diversity in ectoparasite populations: Coevolutionary patterns in Apodemus mice and their lice
Source: Ecol Evol. 2018 Oct 3;8(20):10008–22. doi: 10.1002/ece3.4424 (PMC6206178; doi:10.1002/ece3.4424)
Supplement: Supplementary file 3 [file ECE3-8-10008-s003.pdf]

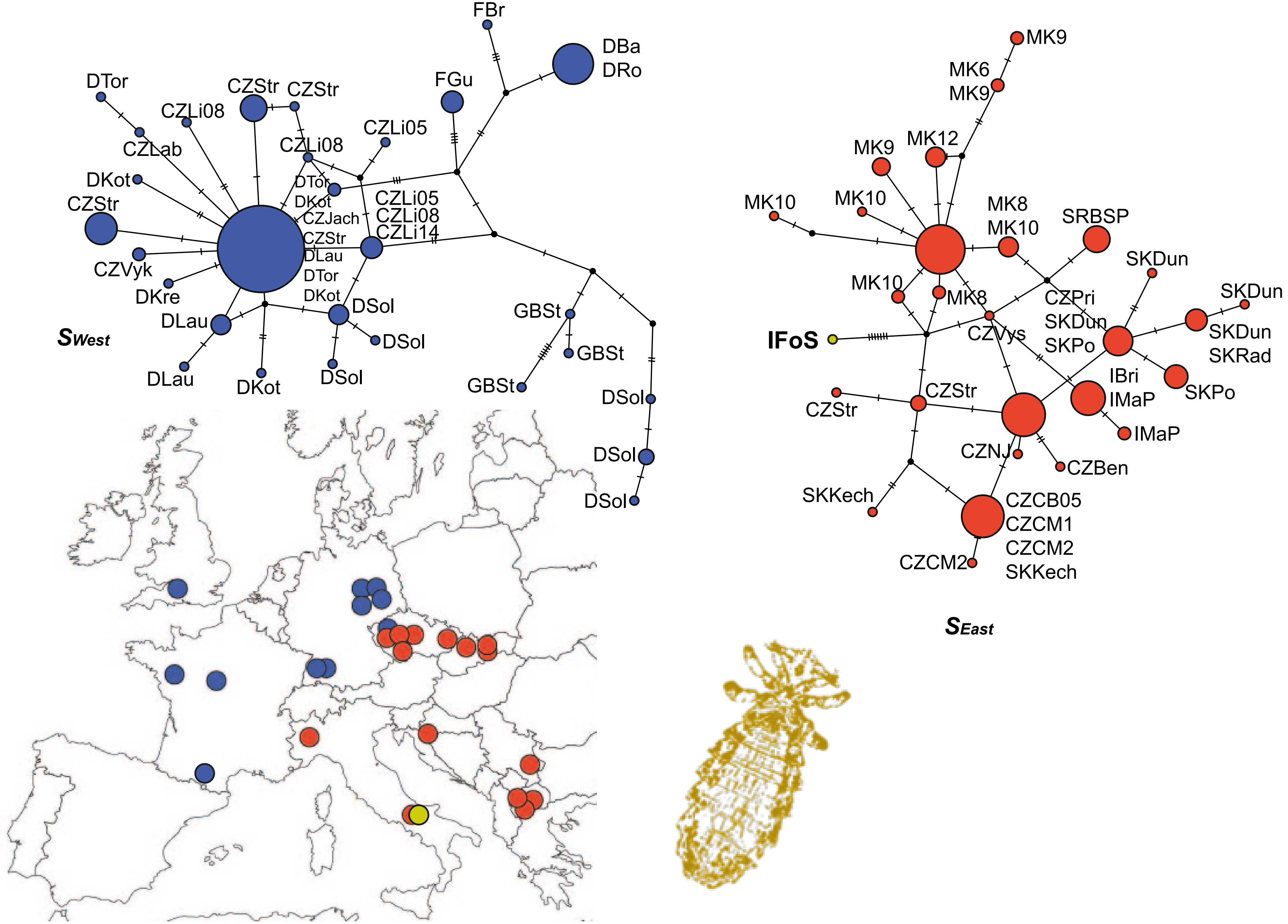

Figure S3: Haplotype networks and distribution map of the *S* lineage of *Polyplax serrata*. Abbreviations: *SWest* – Specific West cluster; *SEast* – Specific East cluster; abbreviations of localities as in Table S1.
