# Supplementary material for: Host specificity driving genetic structure and diversity in ectoparasite populations: Coevolutionary patterns in Apodemus mice and their lice
Source: Ecol Evol. 2018 Oct 3;8(20):10008–22. doi: 10.1002/ece3.4424 (PMC6206178; doi:10.1002/ece3.4424)
Supplement: Supplementary file 4 [file ECE3-8-10008-s004.pdf]

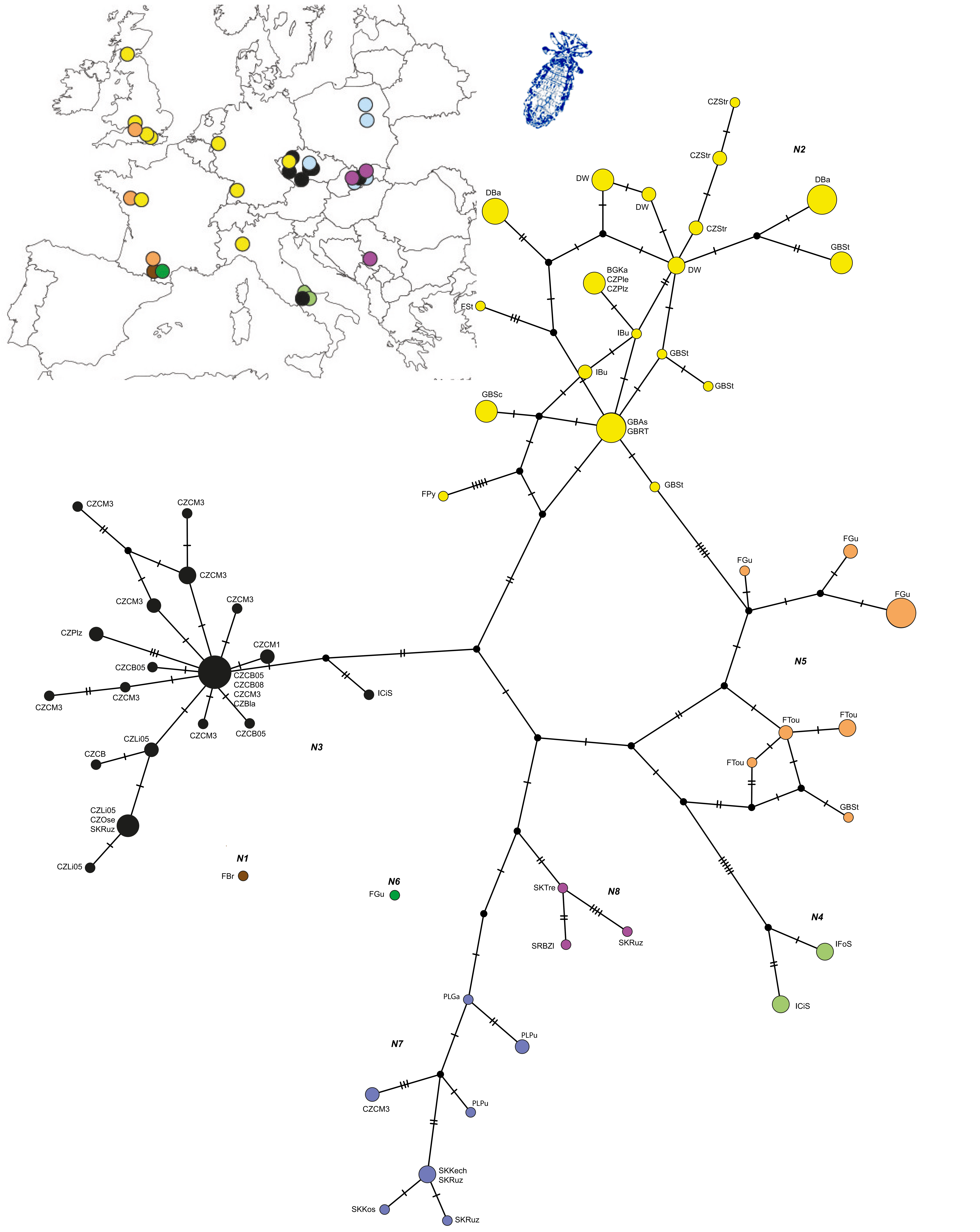

Figure S4: Haplotype network and distribution map of the *N* lineage of *Polyplax serrata*. Colour code for individual subclades (*N1* to *N8*) as in Fig. 2.
