# Supplementary material for: Host specificity driving genetic structure and diversity in ectoparasite populations: Coevolutionary patterns in Apodemus mice and their lice
Source: Ecol Evol. 2018 Oct 3;8(20):10008–22. doi: 10.1002/ece3.4424 (PMC6206178; doi:10.1002/ece3.4424)
Supplement: Supplementary file 6 [file ECE3-8-10008-s006.pdf]

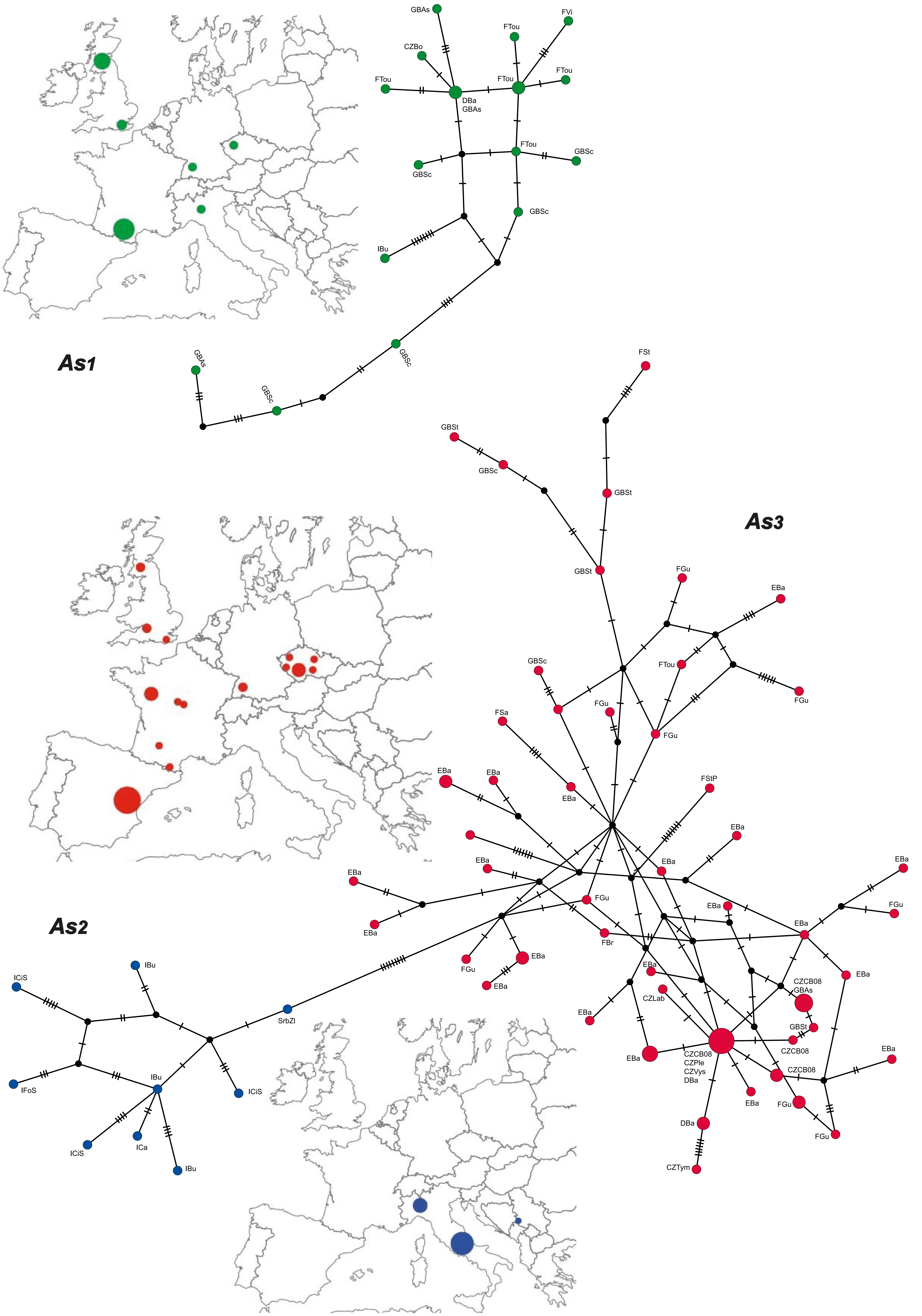

Figure S6: Haplotype networks and geographic distribution of the *Apodemus sylvaticus* subclades *As1*, *As2* and *As3*. Networks were obtained in TCS program implemented in PopArt software using 1002 bp fragments of the mitochondrial D-loop. Abbreviations of geographic localities of haplotypes as in Table S1.
