# Supplementary material for: Host specificity driving genetic structure and diversity in ectoparasite populations: Coevolutionary patterns in Apodemus mice and their lice
Source: Ecol Evol. 2018 Oct 3;8(20):10008–22. doi: 10.1002/ece3.4424 (PMC6206178; doi:10.1002/ece3.4424)
Supplement: Supplementary file 7 [file ECE3-8-10008-s007.pdf]

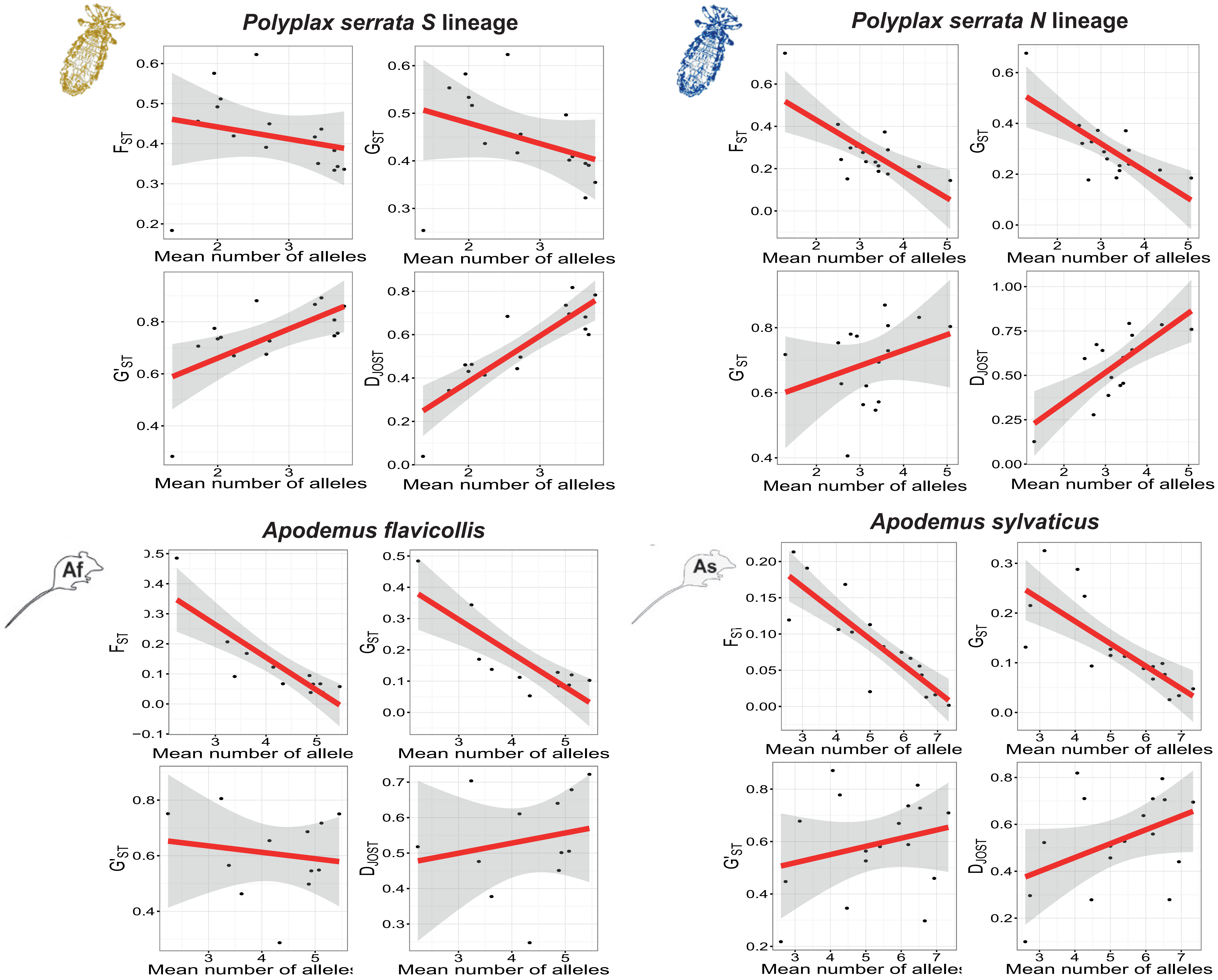

Figure S7: Interaction between F_{ST} statistics (and its derivates G_{ST}, G'_{ST} and D_{JOST}) and the mean number of shared alleles between *Polyplax serrata* of the *S* and *N* lineages, *Apodemus flavicollis and A. sylvaticus* populations.
