# Supplementary material for: Host specificity driving genetic structure and diversity in ectoparasite populations: Coevolutionary patterns in Apodemus mice and their lice
Source: Ecol Evol. 2018 Oct 3;8(20):10008–22. doi: 10.1002/ece3.4424 (PMC6206178; doi:10.1002/ece3.4424)
Supplement: Supplementary file 8 [file ECE3-8-10008-s008.pdf]

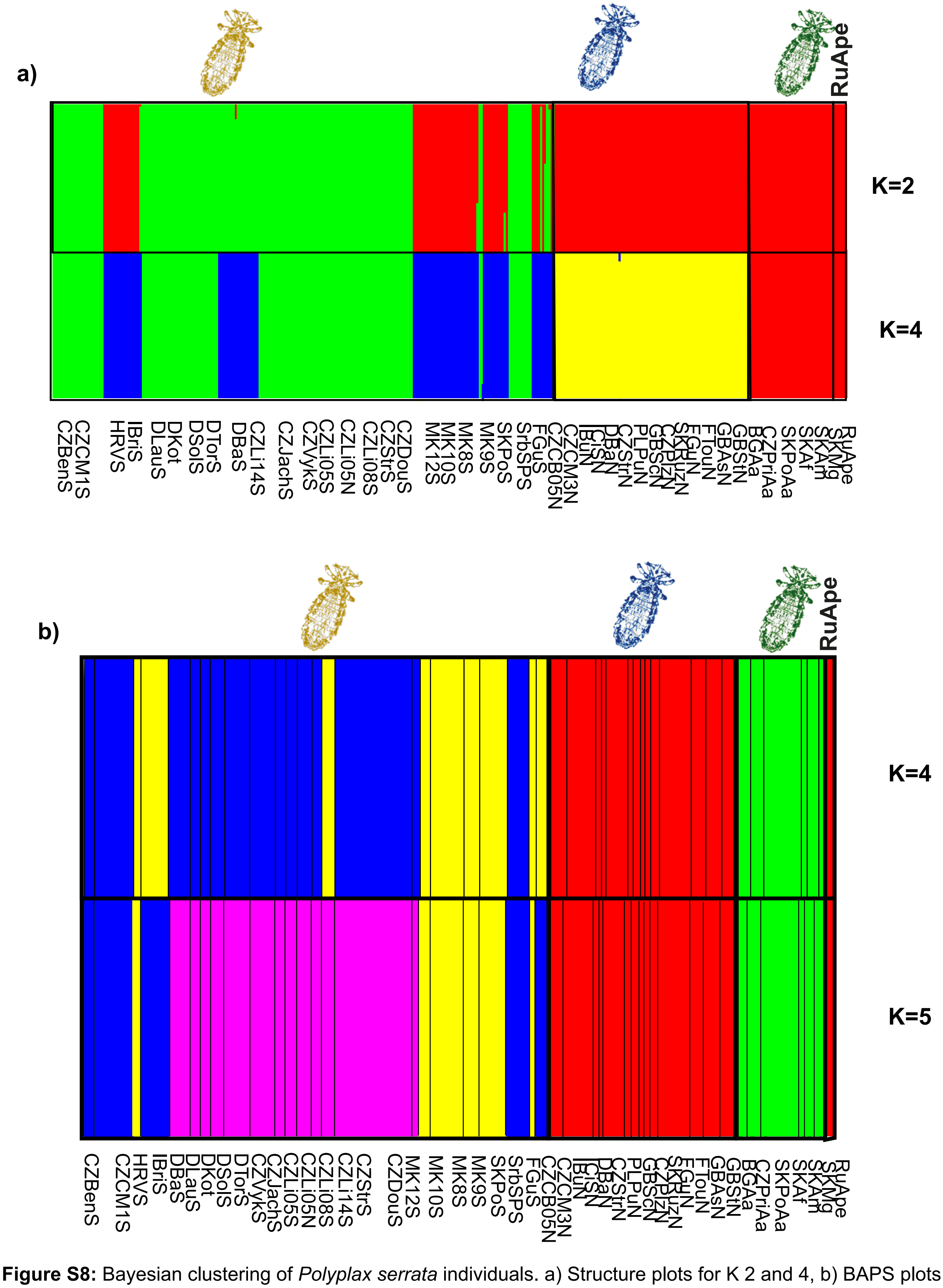

Figure S8: Bayesian clustering of *Polyplax serrata* individuals. a) Structure plots for K 2 and 4, b) BAPS plots for K4 and 5. Yellow louse image –mitochondrial *S* lineage, blue – *N* lineage, green – *Aa* lineage, *RuApe* – lineage from Baikal Lake (*Apodemus peninsulae* host). Abbreviations of populations as in Table S1.
