# Supplementary material for: Host specificity driving genetic structure and diversity in ectoparasite populations: Coevolutionary patterns in Apodemus mice and their lice
Source: Ecol Evol. 2018 Oct 3;8(20):10008–22. doi: 10.1002/ece3.4424 (PMC6206178; doi:10.1002/ece3.4424)
Supplement: Supplementary file 9 [file ECE3-8-10008-s009.pdf]

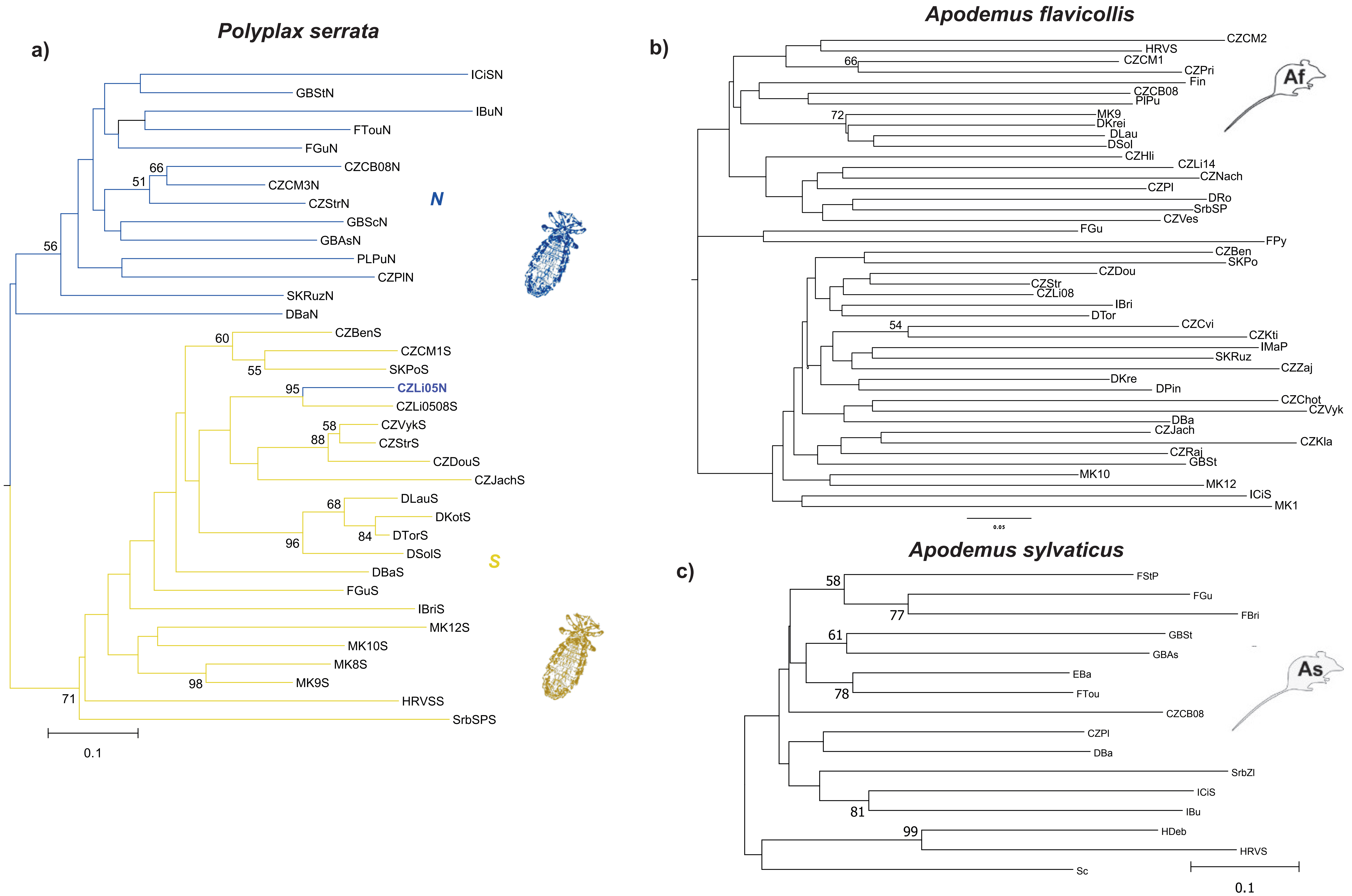

Figure S9: Neighbor-joining trees for *Polyplax serrata* S and N clades (a), *Apodemus flavicollis* (b) and A. *sylvaticus* (c) populations obtained in POPTREEW using pairwise DA values (Nei's genetic distances) calculated from microsatellite data. Bootstrap supports were obtained with 1000 permutations, only values above 50% are shown. Population sample containing mtDNA introgressed from the N lineage of *P. serrata* (CZLi05N) is highlighted in blue. Population abbreviations as in Table S1.
