# Supplementary material for: Host specificity driving genetic structure and diversity in ectoparasite populations: Coevolutionary patterns in Apodemus mice and their lice
Source: Ecol Evol. 2018 Oct 3;8(20):10008–22. doi: 10.1002/ece3.4424 (PMC6206178; doi:10.1002/ece3.4424)
Supplement: Supplementary file 10 [file ECE3-8-10008-s010.pdf]

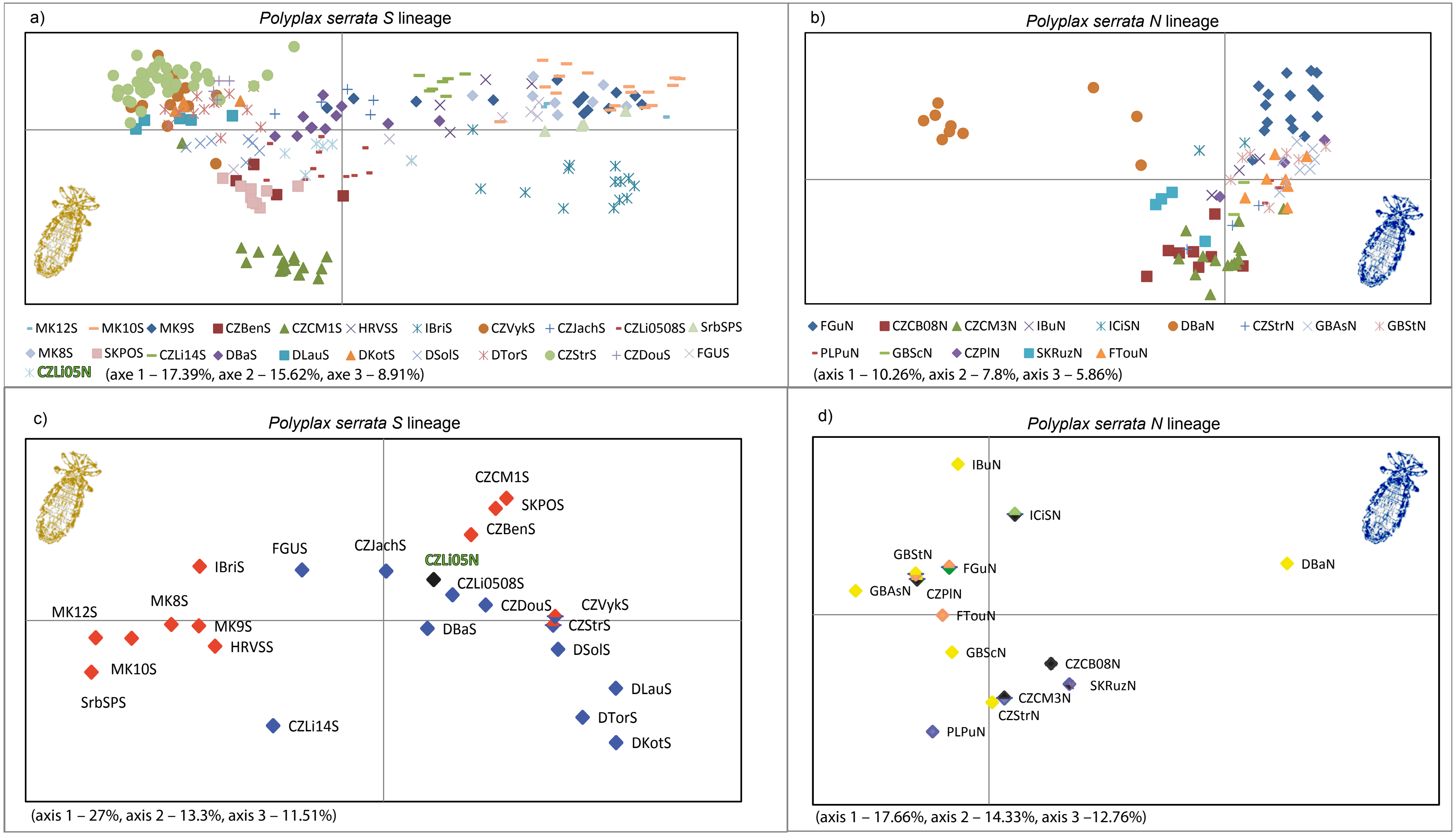

Figure S10: PCoA of *Polyplax serrata* individuals a) and b) and populations c) and d) belonging to *S* and *N* clades (respectively) using microsatellite data. Colours in c) and d) match major lineages used in Fig. 2. Specimens containing mtDNA introgressed from the *N* lineage (CZLi05N) are highlighted in green. Population abbreviations as in Table S1.
