# Supplementary material for: Host specificity driving genetic structure and diversity in ectoparasite populations: Coevolutionary patterns in Apodemus mice and their lice
Source: Ecol Evol. 2018 Oct 3;8(20):10008–22. doi: 10.1002/ece3.4424 (PMC6206178; doi:10.1002/ece3.4424)
Supplement: Supplementary file 11 [file ECE3-8-10008-s011.pdf]

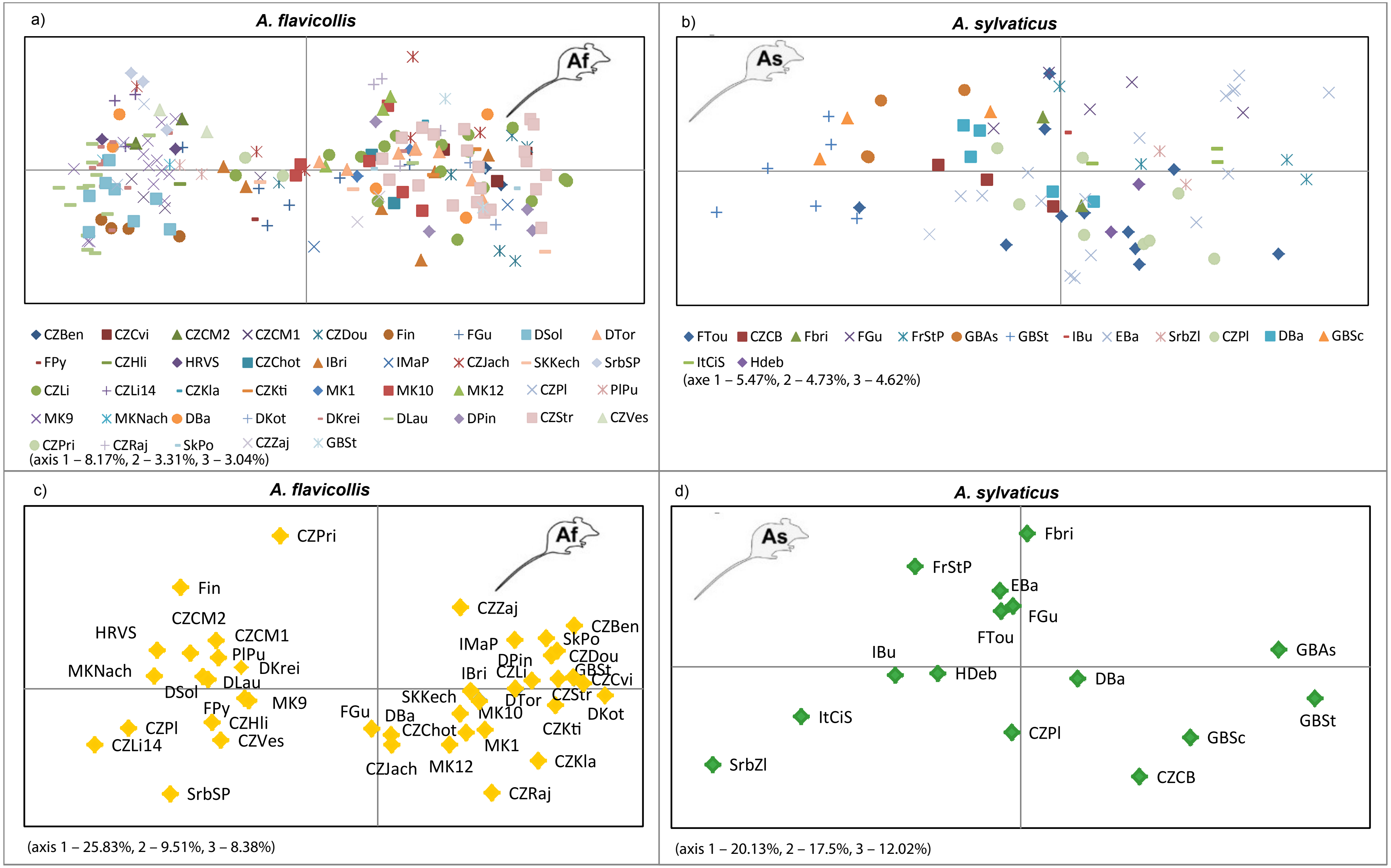

Figure S11: PCoA of *Apodemus flavicollis* a) and *A. sylvaticus* b) individuals and populations c) and d) (respectively) using microsatellite data. Population abbreviations as in Table S1.
