# Supplementary material for: Host specificity driving genetic structure and diversity in ectoparasite populations: Coevolutionary patterns in Apodemus mice and their lice
Source: Ecol Evol. 2018 Oct 3;8(20):10008–22. doi: 10.1002/ece3.4424 (PMC6206178; doi:10.1002/ece3.4424)
Supplement: Supplementary file 12 [file ECE3-8-10008-s012.pdf]

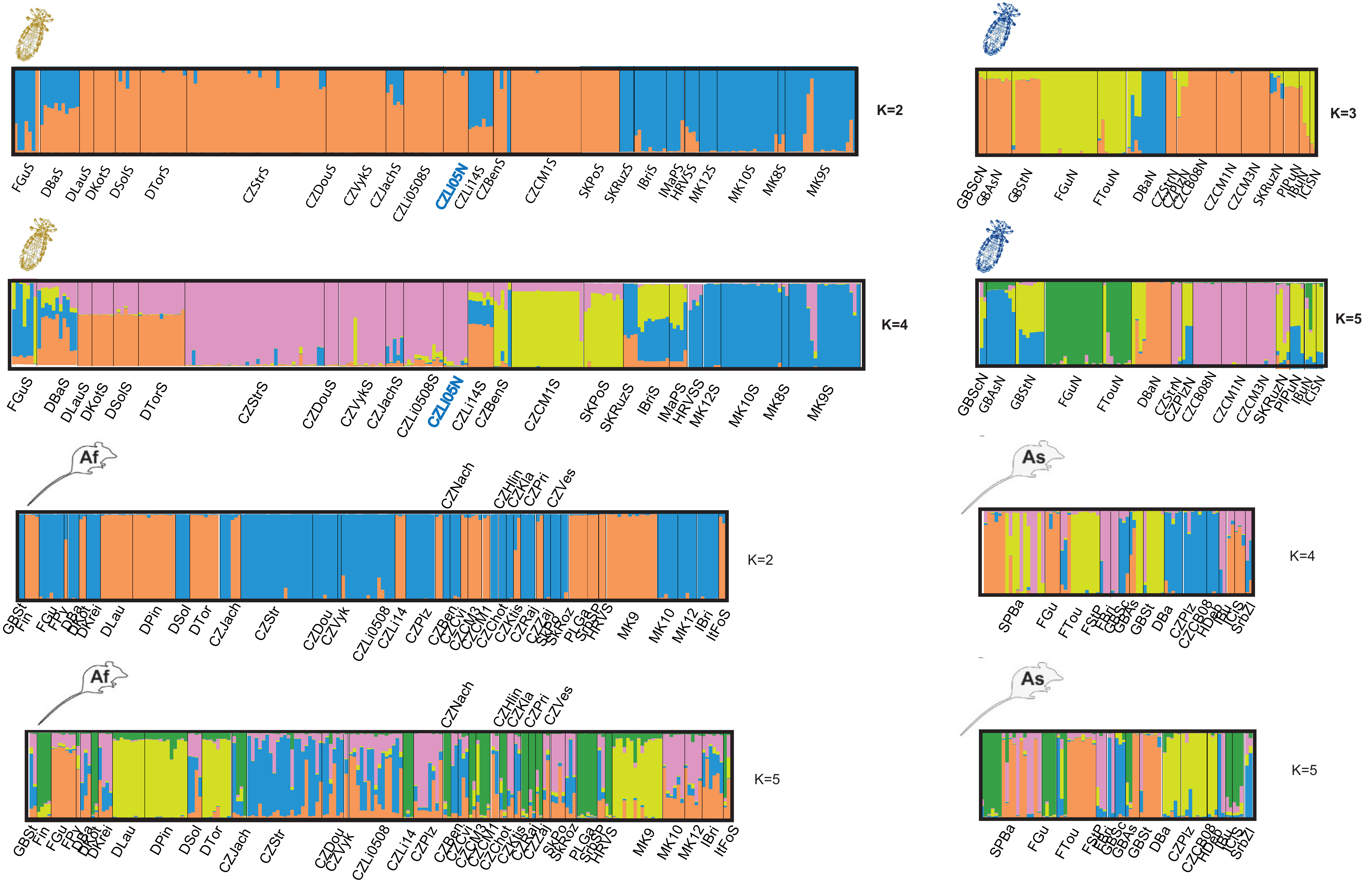

Figure S12: Structure plots for *Polyplax serrata S* lineage (yellow louse image, K2 and 4), *P. serrata N* lineage (blue louse image, K3 and 5), for *Apodemus flavicollis* (Af, K2 and 5) and *A. sylvaticus* (As, K4 and 5). Plots for K values represented by the two highest scores of ‘H are provided. Population containing mtDNA introgressed from the *N* lineage (CZLi05N) is highlighted in blue. Population abbreviations as in Table S1.
