# Supplementary material for: Host specificity driving genetic structure and diversity in ectoparasite populations: Coevolutionary patterns in Apodemus mice and their lice
Source: Ecol Evol. 2018 Oct 3;8(20):10008–22. doi: 10.1002/ece3.4424 (PMC6206178; doi:10.1002/ece3.4424)
Supplement: Supplementary file 13 [file ECE3-8-10008-s013.pdf]

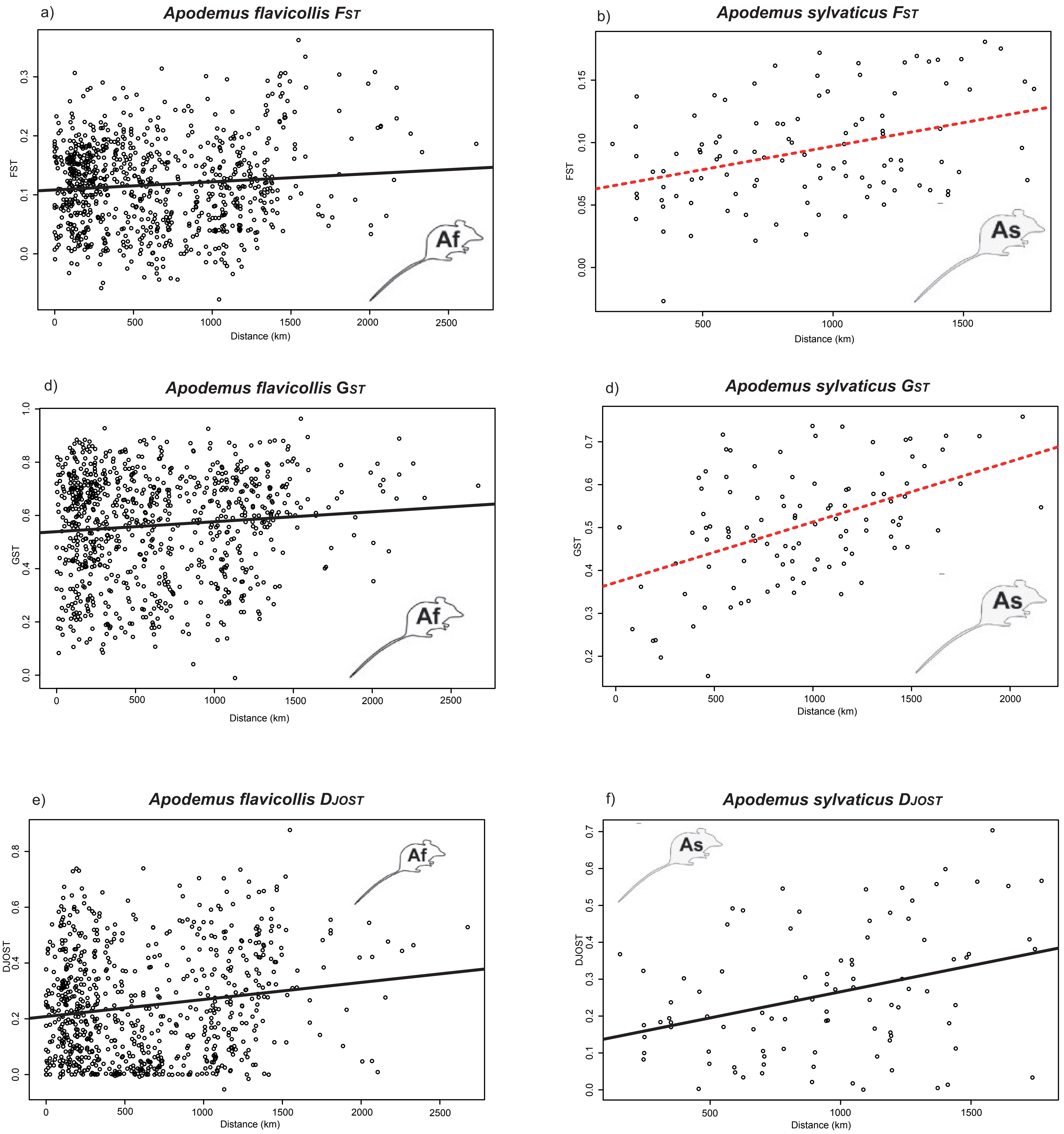

Figure S14: Mantel tests for correlation between genetic diversity indices (F_{ST}, G_{ST} and D_{JOST}) and geographic distance for the populations of *Apodemus flavicollis* (a,c and e) and *A. sylvaticus* (b, d and f) are marked by black (nonsignificant) or red dashed (significant) line. Correlations are significant (P<0.05) for b) F_{ST} and d) G_{ST} of *A. sylvaticus.*
