# Supplementary material for: Host specificity driving genetic structure and diversity in ectoparasite populations: Coevolutionary patterns in Apodemus mice and their lice
Source: Ecol Evol. 2018 Oct 3;8(20):10008–22. doi: 10.1002/ece3.4424 (PMC6206178; doi:10.1002/ece3.4424)
Supplement: Supplementary file 14 [file ECE3-8-10008-s014.pdf]

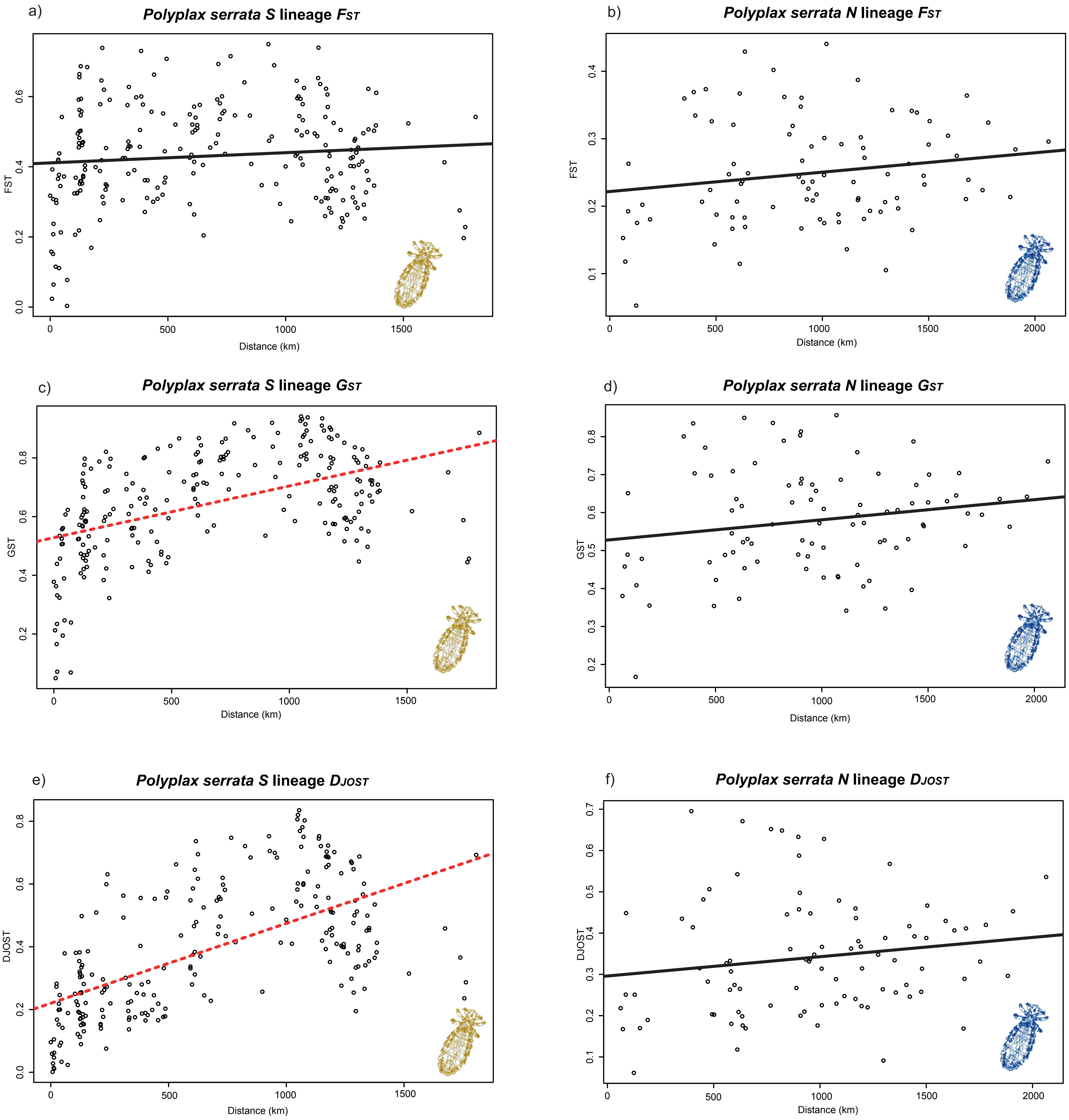

Figure S13: Isolation by distance in the populations of *Polyplax serrata S* (a, c, e in yellow) and *N* lineages (b, d and f in blue). Mantel tests for correlation between pairwise F_{ST} (a and b), G_{ST} (c and d) and D_{JOST} (e and f) indices and geographical distances are marked by black (nonsignificant) or red dashed (significant) line. Correlations are significant ($P<0.05$) for c) G_{ST} and e) D_{JOST} of *Polyplax serrata S*.
