# Supplementary material for: Host specificity driving genetic structure and diversity in ectoparasite populations: Coevolutionary patterns in Apodemus mice and their lice
Source: Ecol Evol. 2018 Oct 3;8(20):10008–22. doi: 10.1002/ece3.4424 (PMC6206178; doi:10.1002/ece3.4424)
Supplement: Supplementary file 15 [file ECE3-8-10008-s015.pdf]

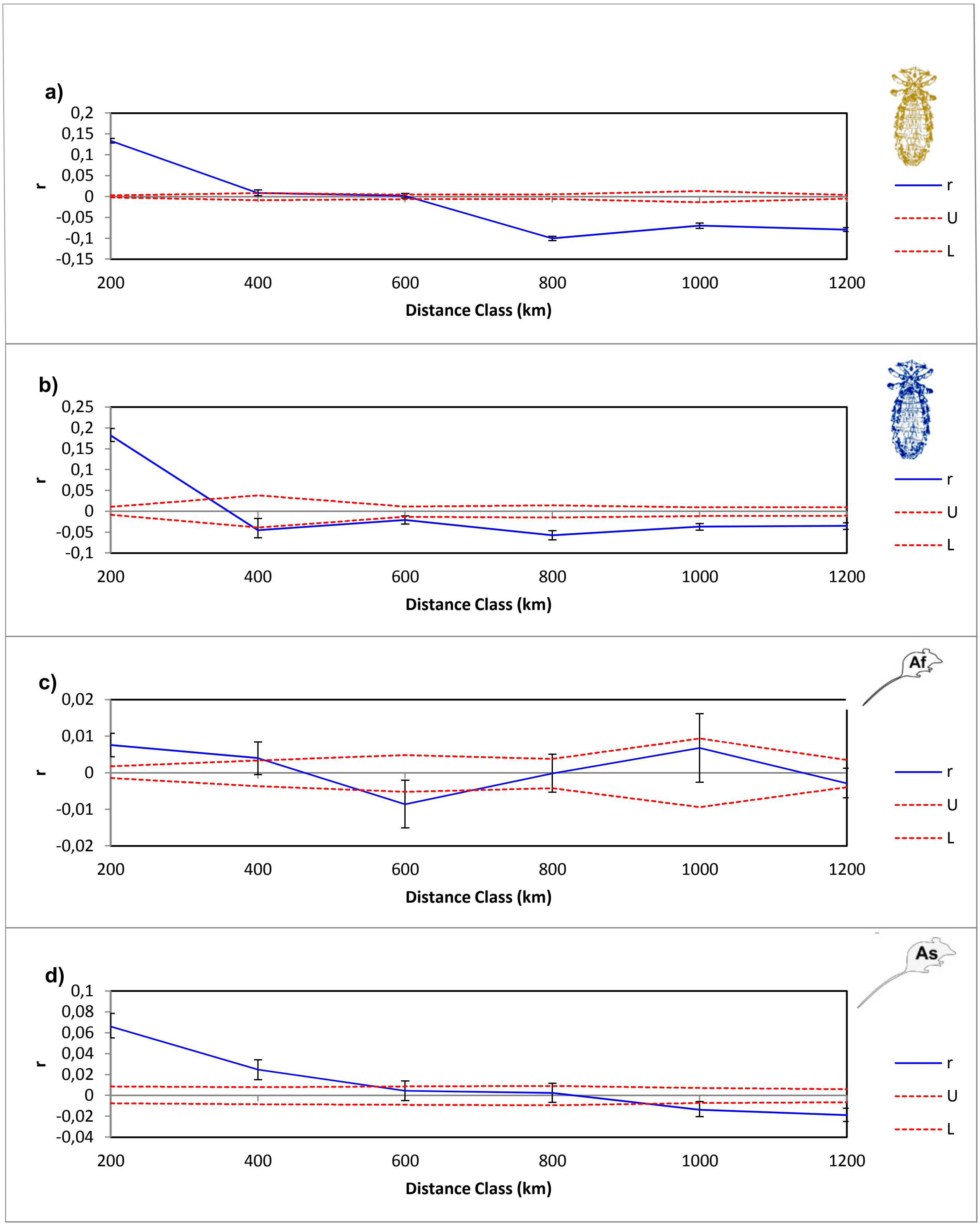

Figure S15: Plots of genetic autocorrelation coefficient (r) across increasing geographic distance class sizes for *Polyplax serrata S* lineage a), *Polyplax serrata N* lineage b), *Apodemus flavicollis* c) and *A. sylvaticus* d). Error bars indicate the 95% confidence interval around the observed r values, and red dashed lines mark the upper (U) and lower (L) 95% confidence interval for the null hypothesis of no spatial autocorrelation (r = 0).
