# Supplementary material for: Host specificity driving genetic structure and diversity in ectoparasite populations: Coevolutionary patterns in Apodemus mice and their lice
Source: Ecol Evol. 2018 Oct 3;8(20):10008–22. doi: 10.1002/ece3.4424 (PMC6206178; doi:10.1002/ece3.4424)
Supplement: Supplementary file 5 [file ECE3-8-10008-s005.pdf]

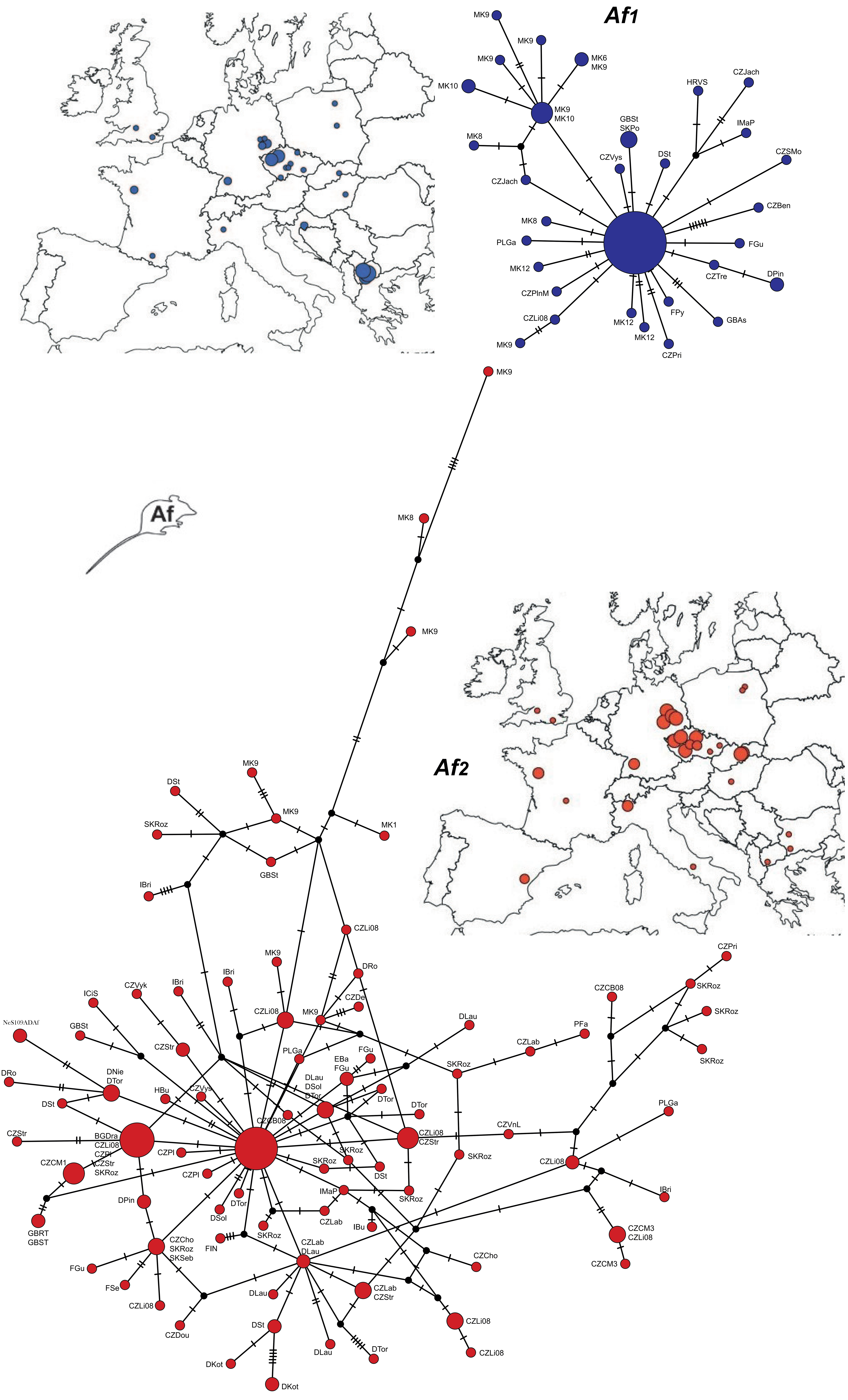


**Figure S5:** Haplotype networks and geographic distribution of the *Apodemus flavicollis* subclades $Af_1$ and $Af_2$. Networks were obtained in TCS program implemented in PopArt software using 1002 bp fragments of the mitochondrial D-loop.

$Af_1$ - EBa, NeRo, CZDou, CZZaj, CZBen, SKRoz, BGSo, DNie, DLau, DSol, DTor

$Af_2$- IMaP, FGu, DRo, DBa, Dlau, DNie, DSol, DPin, DTor, CZLi08, CZJach, CZDou, CZStr, CZPnM, SKPo, PLPu, HDeb
